# The DNA history of a lonely oak: *Quercus humboldtii* phylogeography in the Colombian Andes

**DOI:** 10.1002/ece3.7529

**Published:** 2021-05-07

**Authors:** Sofía Zorrilla‐Azcué, Antonio González‐Rodríguez, Ken Oyama, Mailyn A. González, Hernando Rodríguez‐Correa

**Affiliations:** ^1^ Escuela Nacional de Estudios Superiores (ENES) Unidad Morelia Universidad Nacional Autónoma de México (UNAM) Morelia Mexico; ^2^ Posgrado en Ciencias Biológicas Universidad Nacional Autónoma de México (UNAM) Coyoacán Mexico; ^3^ Instituto de Investigaciones en Ecosistemas y Sustentabilidad Universidad Nacional Autónoma de México (UNAM) Morelia Mexico; ^4^ Laboratorio de Genética de la Conservación Instituto de Investigación de Recursos Biológicos Alexander von Humboldt Bogotá Colombia

**Keywords:** genetic diversity and structure, historical connectivity, historical demography, Neotropical trees, oaks, phylogeography, Pleistocene, *Quercus*

## Abstract

The climatic and geological changes that occurred during the Quaternary, particularly the fluctuations during the glacial and interglacial periods of the Pleistocene, shaped the population demography and geographic distribution of many species. These processes have been studied in several groups of organisms in the Northern Hemisphere, but their influence on the evolution of Neotropical montane species and ecosystems remains unclear. This study contributes to the understanding of the effect of climatic fluctuations during the late Pleistocene on the evolution of Andean mountain forests. First, we describe the nuclear and plastidic DNA patterns of genetic diversity, structure, historical demography, and landscape connectivity of *Quercus humboldtii*, which is a typical species in northern Andean montane forests. Then, these patterns were compared with the palynological and evolutionary hypotheses postulated for montane forests of the Colombian Andes under climatic fluctuation scenarios during the Quaternary. Our results indicated that populations of *Q. humboldtii* have high genetic diversity and a lack of genetic structure and that they have experienced a historical increase in connectivity from the last glacial maximum (LGM) to the present. Furthermore, our results showed a dramatic reduction in the effective population size followed by an expansion before the LGM, which is consistent with the results found by palynological studies, suggesting a change in dominance in Andean forests that may be related to ecological factors rather than climate change.

## Graphical abstract

This study describes the nuclear and plastidic DNA patterns of genetic diversity, structure, historical demography, and landscape connectivity of *Quercus humboldtii*. We found a high population genetic diversity, lack of genetic structure, and evidence of a demographic expansion predating the LGM

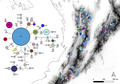

## 1 INTRODUCTION

The study of evolutionary processes in Nearctic and Neotropical montane ecosystems has been a major topic in recent literature. Several groups, such as birds, mammals, and plants, have been used as models to document the role of climatic fluctuations, vulcanism, and tectonics on species evolution, mostly in the Mexican transition zone of the Neotropics (Guevara, 2020; Gutiérrez‐García & Vázquez‐Domínguez, 2013; Ornelas et al., 2013). Such studies have described common phylogeographic patterns among species in response to changes in the environment and have postulated several hypotheses regarding historical population dynamics in the region (Mastretta‐Yanes et al., 2015; Ramírez‐Barahona & Eguiarte, 2013). Despite these advances, emblematic mountainous ranges of the region such as the Andes are apparently less studied.

Tree species play an important role among the variety of biological groups used as models for the study of biogeographic processes. From the Nearctic to the Neotropics, genera such as *Quercus* have been intensively studied from macro‐ and microevolutionary perspectives, allowing us to understand not only how Nearctic lineages have diversified and radiated into the Neotropics (Hipp et al., 2014, 2018, 2019) but also how climatic and geological features have shaped species distributions (Peñaloza‐Ramírez et al., 2020 and references therein). However, there are still few phylogeographic studies of trees distributed in the Neotropical montane forests.

The Andean region, particularly the northern Andes, is a global biodiversity hotspot which harbors some of the highest numbers of endemic plant and vertebrate species in the world (Myers et al., 2000). Despite considerable research effort in scientific areas such as paleoecology and palynology, the evolutionary processes that led to this diversity and its current distribution patterns are not completely understood (Hazzi et al., 2018). In the northern Andes, *Quercus humboldtii* Bonpl. (1805) is a characteristic element of montane ecosystems and the single representative of the genus. Interestingly, numerous paleoecological studies of montane forest evolution during the Quaternary, including the role of *Quercus* in forest dynamics, have been conducted in this region (Hooghiemstra & van der Hammen, 2004 and references therein).

**FIGURE 1:**
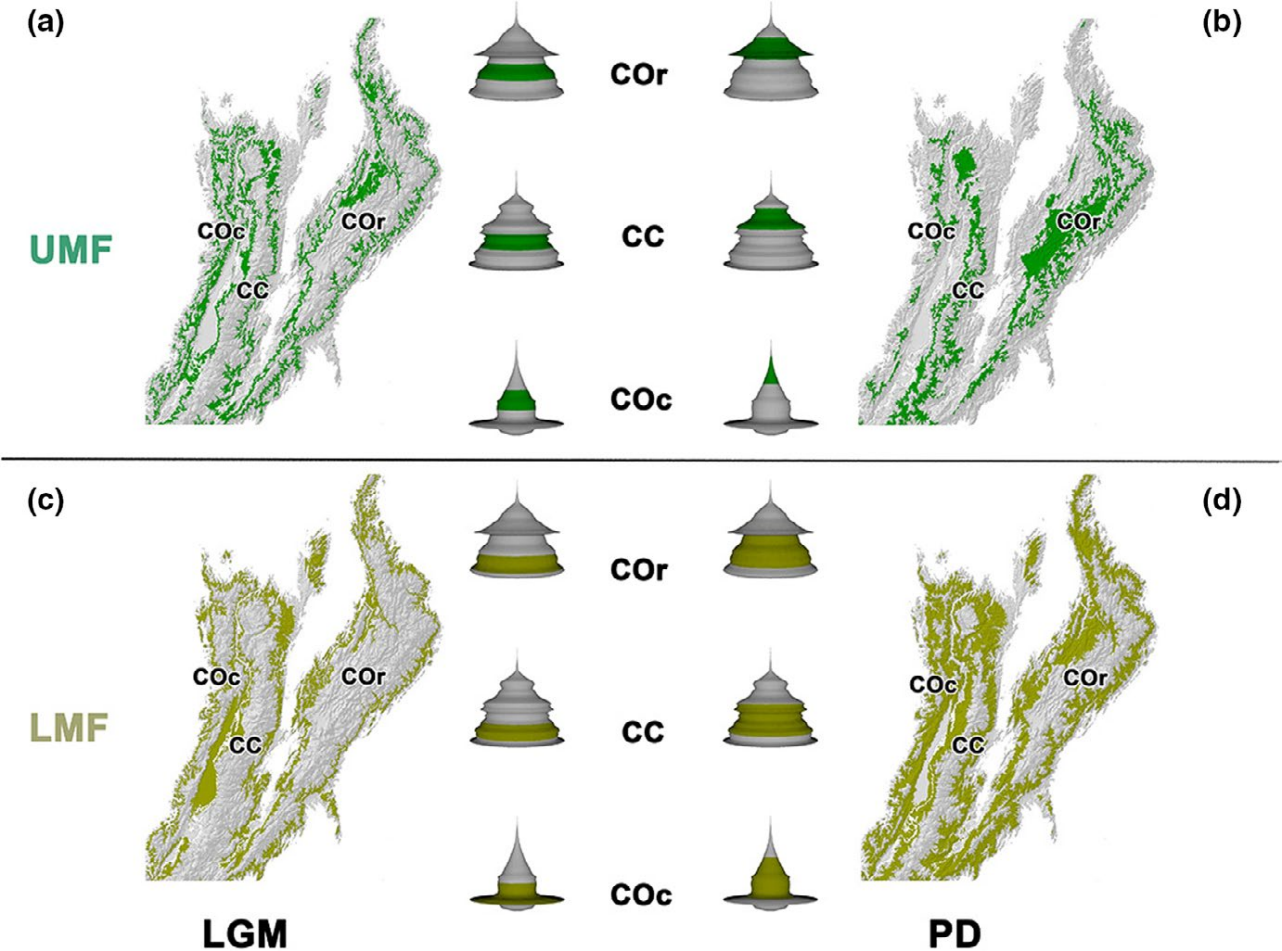
Graphical representation of the historical changes in altitudinal ranges for the upper montane forest (UMF; a and b) and lower montane forest (LMF; c and d) (Hooghiemstra & Flantua, 2019). Columns represent altitudinal ranges for the last glacial maximum (LGM; a and c) and present day (PD; b and d). Next to each map, in the middle columns, is shown three‐dimensional solid of revolution of the variation in superficial area (*x*‐ and *y*‐axis) along elevation (*z*‐axis) for each of the main Colombian Andes cordilleras. Green color represents the geographic altitudinal range occupied by vegetation belts (dark green UMF; light green LMF) during each period. Superficial area was calculated for elevation bins of 100 m

Recently, Hooghiemstra and Flantua (2019) reviewed the studies of northern Andean fossil pollen records for the upper and the lower montane forests (UMF and LMF, respectively), which are the biomes where *Q. humboldtii* is distributed (Hooghiemstra, 2006; Rangel‐Ch & Avella, 2011). They describe that during the last glacial maximum (21 ka BP to ~14 ka BP), a period characterized as extremely cold and dry (van der Hammen, 1974), the upper forest limit reached an elevation of ~2,000 m (van der Hammen & Cleef, 1986), and the UMF was displaced to a lower elevation and compressed by ~400 m compared to today's altitudinal range (Figure 1a,b). However, because of the Andean topography, the available surface area for the UMF varies only slightly (2.4%) between the LGM and the present (Hooghiemstra & Flantua, 2019). The LMF was also displaced altitudinally to a range of less than half the vertical span of the current altitudinal distribution. But, in contrast with the UMF, this vertical compression (~700 m) resulted in a reduction of ~42% of the superficial area (Hooghiemstra & Flantua, 2019) (Figure 1c,d). Following ~14 ka BP, the global temperatures became less cold, and the upper forest limit migrated upslope to eventually reach its current position (3,200–3,400 m) (Hooghiemstra & van der Hammen, 2004). These fluctuations probably had different consequences on the demography and genetics of the species that constitute the Andean montane forests.

The altitudinal changes in vegetation belts in this heterogeneous topographical context may have caused gene flow and connectivity to change among populations. Several phylogenetic studies have drawn attention to the importance of the mid‐ and late Pleistocene periods for highland species diversification, identifying lowlands, river valleys, and some ridges as important geologic features that may have acted as barriers and favored species diversification. However, distribution patterns can be more complex, and some species have crossed these barriers (Cadena et al., 2007; Muñoz‐Ortiz et al., 2014), suggesting long‐distance dispersal or the transience of barriers (Sanín et al., 2017). Specifically, for *Q. humboldtii*, previous palynological and floristic studies have proposed migration paths that allowed this species to colonize different mountain ranges (van der Hammen, 2008; Rangel‐Ch & Avella, 2011). Although these routes were proposed in the context of colonization, they can be seen as possible ways in which the oak forests remained connected over time; therefore, populations of *Q. humboldtii* are expected to have maintained gene flow.

A few models have been recently proposed to describe the vegetation history within Neotropical mountainous regions. Each focuses on different factors that might have shaped recent evolutionary processes, such as the joint effect of precipitation and temperature (Ramírez‐Barahona & Eguiarte, 2013), the interaction of climate, topography, and volcanism at a microevolutionary scale (Mastretta‐Yanes et al., 2015), and the effect of the interaction of fluctuating climate and topography on the degree of historical connectivity from a macroevolutionary perspective (Flantua & Hooghiemstra, 2018; Hazzi et al., 2018). These models are hypotheses against which phylogeographic data can be compared to improve the knowledge of general evolutionary processes in these landscapes.

As far as we know, there are no phylogeographic studies that evaluate the effects of these range shifts on historical population sizes and connectivity for UMF and LMF tree species, which contrasts with the growing number of studies evaluating phylogenetics and phylogeography of species of the Andean paramo (Flantua et al., 2019). Moreover, the abundance of palynological data makes the Andes an optimal scenario for testing historical demography hypotheses about the effect of historical climatic changes on the elements of the montane forest in the Neotropics. Thus, this study aims to evaluate the effects of the climatic fluctuations that have occurred since the last glacial maximum on the population history of *Q. humboldtii*, a typical element of the northern Andean Montane Forest. Specifically, we described the nuclear and plastidic patterns of genetic diversity and genetic structure. We analyzed the historical demography and landscape connectivity of this species. Finally, we compared our results with the palynological and evolutionary hypotheses postulated for montane forest dynamics in the Colombian Andes under climatic fluctuation scenarios during the Quaternary.

## 2 METHODS

### 2.1 Study species

**FIGURE 2:**
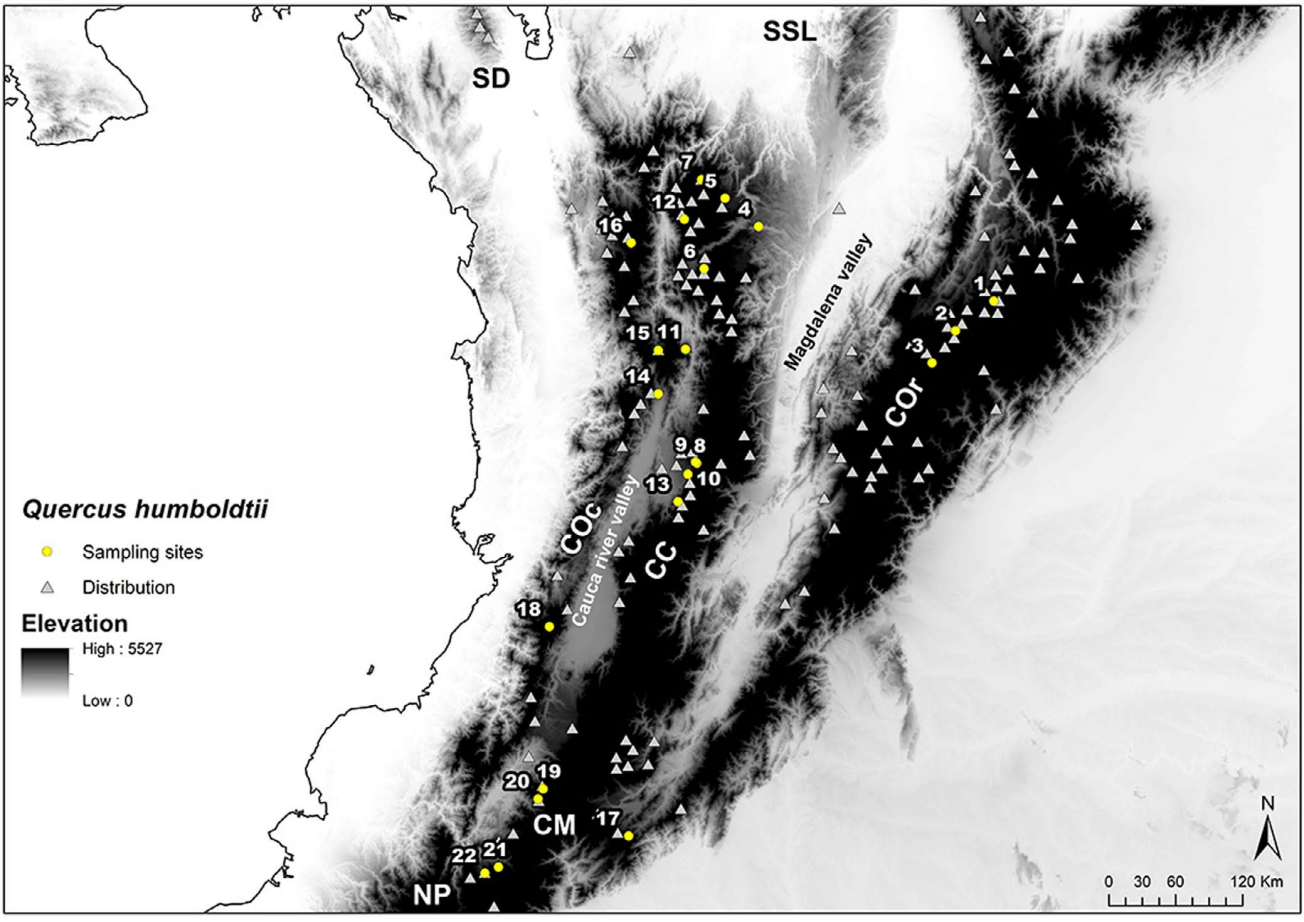
*Quercus humboldtii* distribution is represented by occurrence records (gray triangles) and sampling locations included in this study (yellow circles). In the background, elevation is displayed in a grayscale where light colors correspond to lowlands and darker colors to highlands. COc, Cordillera Occidental; CC, Cordillera Central; COr, Cordillera Oriental; CM, Macizo Colombiano; SD, Serranía del Darién; SSL, Serranía de San Lucas; NP, Nudo de los Pastos

*Quercus humboldtii* (Bonpl. 1805) is a large tree that produces mid‐sized acorns (2–3 cm long) annually and has evergreen lanceolate leaves (Müller, 1942). It is distributed in northern South America (Figure 2), from the Colombian Darién Gap south to the Nariño region in southern Colombia through the Andean Mountains, in an altitudinal range from 774 to 3,200 m (Pulido et al., 2006; Rangel‐Ch & Avella, 2011). Generally, oak forests in Colombia occur in areas with a mean annual temperature range of 10–13°C, a thermal limit at 24°C, and precipitation up to 2,800 mm (Avella et al., 2017). Andean and sub‐Andean oak forests are formed by multiple phytosociological associations. However, they are generally characterized by the dominance of tree strata, particularly *Q. humboldtii* (Pulido et al., 2006).

### 2.2 Sampling and molecular protocols

*Quercus humboldtii* leaf tissue representing a total of 22 sampling locations (Table S1; Figure 2), with between five and 13 samples from each location, was provided by the IAvH Tissue Collection (http://www.humboldt.org.co/es/investigacion/programas/colecciones‐biologicas/tejidos) for the molecular procedures. Total DNA was extracted using a commercial DNA isolation kit under standard protocols (QIAGEN DNeasy Plant Mini Kit). A set of nine mononucleotide repeat chloroplast DNA microsatellite (cpSSR) loci (cmcs2, cmcs3, cmcs4, cmcs5, cmcs6, cmcs7, cmcs10, cmcs12, and cmcs14) and 10 nuclear DNA microsatellite (nSSR) loci (quru‐GA‐IF07, ‐IC08, ‐OM07, ‐OE09, ‐2F05, ‐IF02, ‐OC11, ‐OA01, ‐OI01, and ‐OC19) designed for Fagaceae species by Sebastiani et al. (2004) and Aldrich et al. (2002), respectively, were screened and tested for polymorphism to collect genome‐wide information related to pollen‐ and seed‐mediated demographic processes.

Polymerase chain reactions (PCRs) were performed using the QIAGEN multiplex PCR kit with a final volume of 5 μl containing 1X multiplex PCR master mix, 0.25 mM of each primer, 20 ng of DNA, and dH_2_O. Amplification was performed by multiplexing groups of cpSSRs and nSSRs as follows: (i) two primer groups for cpSSRs (the first containing cmcs3, cmcs4, cmcs5, and cmcs6 and the second containing cmcs2, cmcs7, cmcs10, cmcs12, and cmcs14) and (ii) four primer groups for nSSRs (a) quru‐GA‐IF02 and ‐IF07; (b) ‐OE09, ‐OM07, and ‐2F05; (c) ‐OC11, ‐IC08, and ‐OC19; and (d) ‐OI01 and ‐OA01). PCR and fragment sizing were performed following the procedures described by Rodríguez‐Correa et al. (2017), Rodríguez‐Correa et al. (2018).

### 2.3 Molecular data analysis

Each size variant for the evaluated microsatellite loci for both cpSSR and nSSR was defined and manually checked using GeneMarker v2.4.6 (Hulce et al., 2011). Binning was performed using the TANDEM (Matschiner & Salzburger, 2009) and Flexibin (Amos et al., 2007) programs for the nSSR and the cpSSR database, respectively. Nuclear microsatellite loci were tested for the presence of null alleles. First, all individuals were grouped as a single population using Micro‐Checker 2.2.3 (Van Oosterhout et al., 2004) and then for each sampling location separately using FreeNa (Chapuis & Estoup, 2007). Because the estimation of null allele frequency can be influenced by inbreeding and vice versa, the INEST 2.2 (Chybicki & Burczyk, 2009) program was used to estimate inbreeding coefficients and null allele frequency simultaneously. All the possible models were tested using 500,000 cycles, out of which 50,000 were used as burn‐in and 1,000 were kept for calculation of the results. Deviance information criterion (DIC) values for all the models were compared to choose the best model. To evaluate the influence of null alleles on the estimation of the genetic structure, FreeNa (Chapuis & Estoup, 2007) was further used to calculate *F_ST_* with and without the ENA correction. Finally, nuclear loci were tested to detect significant departures from Hardy–Weinberg equilibrium and pairwise linkage disequilibrium among loci using the program Arlequin 3.5.2.2 (Excoffier & Lischer, 2010).

### 2.4 Genetic diversity

For the purpose of describing the genetic variation within populations, haplotype richness, rarefied haplotype richness, genetic diversity with unordered alleles (h sensu Pons & Petit, 1996), and nonstandardized genetic diversity with ordered alleles (v sensu Pons & Petit, 1996) were calculated for the 22 sampling locations (226 individuals) using cpSSR loci and the programs SPAGeDi 1.1 (Hardy & Vekemans, 2002) and Arlequin 3.5.2.2 (Excoffier & Lischer, 2010). The number of alleles (A), effective number of alleles ($A_{e}$), number of private alleles ($A_{p}$), observed ($H_{o}$) and unbiased expected (u $H_{e}$) heterozygosity, and the rarefied allele richness (AR) were calculated for the 22 sampling locations (211 individuals) using nSSR loci and the programs Arlequin 3.5.2.2 (Excoffier & Lischer, 2010) and GenAlEx 6.5 (Peakall & Smouse, 2006, 2012), and the diveRsity (Keenan et al., 2013) package in R (R Core Team, 2019). The previously mentioned genetic diversity estimators were calculated at the population and regional levels (Cordillera Occidental, Cordillera Central and Cordillera Oriental; Figure 2). The differences in the number of genotyped individuals between the cpSSR and nSSR analyses were due to failure in the amplification of several microsatellite loci after different trials in a few specific individuals. Only those with a maximum of one locus of missing data were retained in the nSSR database.

### 2.5 Genetic structure

Analyses of molecular variance (AMOVAs) were performed to describe the partitioning of the genetic variation between groups, between sampling locations within groups, and within sampling locations for both cpSSR and nSSR datasets. The group categories were defined as the regional levels described above, which correspond to the three main cordilleras of the Colombian Andes. The AMOVA was calculated considering both $F_{\mathrm{ST}}$ (based on an infinite allele mutation model, IAM) and $R_{\mathrm{ST}}$ (based on a stepwise mutation model, SMM) using 10,000 permutations in Arlequin 3.5.2.2 (Excoffier & Lischer, 2010).

The grouping of individuals based on their shared genetic information (i.e., genetic structure) was further evaluated considering the geographic location. For this purpose, the package *tess3r* (François et al., 2006) was implemented in R 3.6.1 (R Core Team, 2019) for the analysis of the nSSR dataset. This algorithm is based on the optimization of least squares and the factorization of a genetic distance matrix constrained by geography (Deng et al., 2011; François et al., 2006; Frichot et al., 2014). The algorithm searches for ancestry coefficients of the K genetic groups among individuals, based on the concept that individuals within a close geographic proximity are more likely to share ancestral genotypes (François et al., 2006). The algorithm was run using the function's default parameters for a range of genetic groups (K) from 1 to 22, with 100 replicates for each K. In each run, 5% of the data was masked as missing data and was used to test the predicted results through a cross‐entropy criterion (Frichot et al., 2014). The optimal value of K was determined through the lowest value of the cross‐entropy criterion (RMSE) before it plateaued or increased again (Frichot & François, 2015).

To test for the presence of phylogeographic structure, in the case of cpSSRs, the observed and permuted values of $N_{\mathrm{ST}}$ ($N_{\mathrm{ST}}$ and $pN_{\mathrm{ST}}$, respectively) were compared. *N_ST_* is an $F_{\mathrm{ST}}$ analog that takes into account the genetic distances between haplotypes. The $pN_{\mathrm{ST}}$ value should approximate the value of $G_{\mathrm{ST}}$, which considers only the probability of identity by descent among individuals among populations. Therefore, significant differences between $N_{\mathrm{ST}}$ and $pN_{\mathrm{ST}}$ are indicative of phylogeographic structure, meaning that related haplotypes are located in the same populations (Pons & Petit, 1996). In a similar way, phylogeographic structure can be tested by comparing $R_{\mathrm{ST}}$ and permuted $R_{\mathrm{ST}}$ ($pR_{\mathrm{ST}})$ for nSSRs. $R_{\mathrm{ST}}$ is a SMM‐based measure of differentiation, which is calculated from differences in allele sizes. Permuted $R_{\mathrm{ST}}$ is expected to approximate the value of $F_{\mathrm{ST}}$; therefore, these values can be used to infer the relative importance of stepwise mutation versus drift in population differentiation (Hardy et al., 2003). All of the abovementioned values were calculated using SPAGeDi 1.5 (Hardy & Vekemans, 2002). In addition, a minimum spanning network was computed using Network 5.0.1.1 to infer cpSSR haplotype relationships with a maximum parsimony search (Polzin & Daneshmand, 2003).

### 2.6 Historical demography

**FIGURE 3:**
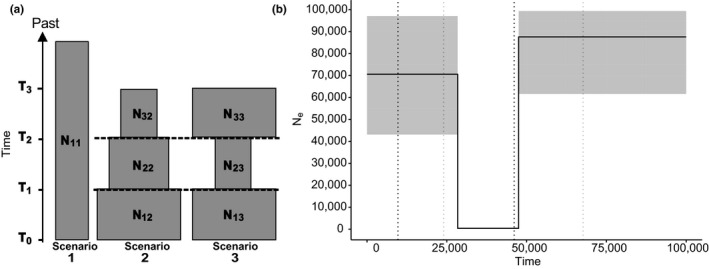
(a) Scheme of three demographic scenarios tested with DIYABC representing the following hypothesis. Scenario 1: constant effective population size, scenario 2: progressive and constant population expansion, and scenario 3: a bottleneck event followed by a recent population expansion (see Table S2 for parameter prior values). Effective population sizes are represented by *N*, the first subscript represents order in time, and the second represents the scenario in which the parameter was used (i.e., $N_{11}$ corresponds to the most recent effective population in scenario 1). Time was represented as *t* with higher values corresponding to events further in the past. (b) Scaled representation of the results from the best scenario (3) obtained from DIYABC analysis. Black solid line represents the mean effective population size through time in years. Shaded area represents 95% confidence intervals for $N_{e}$. Dotted lines indicate 95% confidence intervals for time: black dotted line for the most recent event (expansion) and light gray dotted line for a previous event (bottleneck)

Approximate Bayesian computation was used to test different hypotheses about the historical demography of *Q. humboldtii*. All the sampling locations were considered as a unique population due to the high level of gene flow observed and the lack of genetic groups evidenced by previous analyses. The posterior probabilities of three scenarios were compared with the objective of testing the following hypotheses (Figure 3a): (i) constant effective population size; (ii) progressive and constant population expansion; and (iii) a bottleneck event followed by a recent population expansion. The analysis was implemented in DIYABC 2.1.0 (Cornuet et al., 2014) using the nSSR data. Several short trials were performed to determine the ranges of the prior distributions.

Finally, a total of 3 million datasets were drawn from the priors described in Table S2 to compare with the summary statistics of the observed data, although only a subset of the closest 1% of the datasets was used in the calculation of the posterior probabilities. Loci were grouped and defined by a stepwise mutation model (SMM) with a mutation rate ranging from 1 × 10^–3^ to 1 × 10^–4^ (Cavender‐Bares et al., 2011; Dow & Ashley, 1998; Muir et al., 2004). All the summary statistics available in the program were considered when calculating the posterior probabilities. The scenario that best described the data was chosen based on the frequency of each scenario in the simulated datasets that most closely matched the observed data and a logistic regression approach (Beaumont et al., 2002; Cornuet et al., 2008). The parameter results related to time were translated into years considering a generation time of 100 years (Gugger et al., 2013).

Confidence in DIYABC scenario choice was evaluated in three ways. All the methods are based on simulating test datasets (pseudo‐observed datasets) and estimating their respective posterior probabilities. The first method checks the posterior error rate. It selects the 500 closest datasets to the observed data and uses them as test datasets. Then, it calculates the proportion of times the wrong scenario had the highest posterior probability. The second method uses 1,000 test datasets randomly chosen from the whole prior space of the hypothesized best scenario (X) and calculates the proportion of times in which other scenarios have the highest posterior probability given that scenario X is true (*type I error*). Similarly, the third method calculates the proportion of times in which scenario X has the highest posterior probability given that a different scenario is true. This proportion is calculated for all the other scenarios (*type II error*).

### 2.7 Connectivity analysis

For the purpose of evaluating the geographic connectivity dynamics of *Q. humboldtii* over time, the following general workflow was followed: (i) First, we used ecological niche modeling to build a climatic suitability‐based distribution model for the species through the Colombian Andes in different time periods. (ii) Then, we selected suitable habitat patches for each period. (iii) Subsequently, we constructed a graph using the least‐cost path among patches. (iv) Finally, we calculated and compared the equivalent connectivity area (ECA) among time periods.

The geographic records of *Q. humboldtii* used for the ecological niche modeling (ENM) were gathered from public databases (Global Biological Information Facility; 03 July 2019 https://doi.org/10.15468/dl.cnhmvu), field observations, and bibliographic references. Occurrence records were mapped and filtered by altitude, latitude, and longitude based on the natural history of the species. Finally, to diminish the possible spatial autocorrelation effects of the aggregation of occurrence records on the ENM, all the records except one within a 10 km radius were deleted from the database.

The climatic layers used for the present day (PD) were developed by Fick and Hijmans (2017) (mid‐Holocene (MHol) version 2.0). The last glacial maximum (LGM) climatic layers used were based on three global climate models (GCMs): (i) The Community Climate System Model (CCSM4; Gent et al., 2011); (ii) the Japan Agency for Marine‐Earth Science and Technology, Atmosphere and Ocean Research Institute (The University of Tokyo) and the National Institute for Environmental Studies MIROC‐ESM model (MIROC; Watanabe et al., 2011); and (iii) the Max Planck Institute for Meteorology MPI‐ESM‐P model (MPI; Giorgetta et al., 2013). The climate layers that were used had spatial resolutions of 30 arc seconds (PD and MHol) and 2.5 arc minutes (LGM). To avoid redundancy among the climatic variables during the ENM, a subset of variables was defined using a pairwise correlation test between the variables using R 3.6.1 (R Core Team, 2019). When the values of the correlation were higher than 0.7, the more specific variable of each pair was discarded (e.g., maximum temperature of the warmest month vs. annual mean temperature).

Ecological niche modeling was performed using the maximum entropy algorithm executed in MaxEnt version 3.3.3a (Phillips et al., 2006). The MaxEnt model was implemented using true presence records along the Colombian Andes and five independent climatic variables that remained after the pairwise correlation tests (annual mean temperature, temperature seasonality, temperature annual range, annual precipitation, and precipitation seasonality). Paleodistribution models of *Q. humboldtii* in the Colombian Andes during the MHol and LGM were obtained by projecting the present distribution model into six MHol and LGM scenarios (CCSM, MIROC, and MPI for each period). The models were evaluated using the average AUC (area under the curve) as an independent threshold after a 100‐replication bootstrap procedure. A subset formed by 30% of the total records was used for training the AUC, and the remaining 70% of the data were used to test the AUC.

The connectivity among habitat patches during each period (PD, MHol, and LGM) was evaluated using the graph‐based equivalent connectivity area (ECA) index (Saura et al., 2011). This metric postulates that connectivity occurs inside each habitat patch and among patches and therefore takes into account habitat availability (patch area) and the patch's topological position in a network (i.e., the connections each patch has with the rest of the patches) to obtain the size of a single patch with maximum connectivity that would reflect the same connectivity value as the observed landscape (Saura et al., 2011; Saura & Pascual‐Hortal, 2007).

The calculation of ECA requires two inputs: (i) a collection of suitable habitat patches and (ii) the least‐cost distances among them. To obtain the first input, a resistance layer was constructed using the average MaxEnt output model for each period and the present‐day topographic ruggedness index (TRI), which is the sum of the changes in elevation among neighboring cells (Riley et al., 1999). The niche model for each period was rescaled using a maximum sensitivity test plus specificity logistic threshold to identify the cells with the lowest resistance value, and all the cell values below this threshold were recalculated using the function (100 − (threshold − “value”) * 100/threshold) (Zhang et al., 2019). The topographic ruggedness index was classified into 10 natural breaks using ArcGIS 10.3 (ESRI, 2014) to assign a habitat suitability value to each category.

These two layers were combined into a habitat suitability layer using the Gnarly Landscape Utilities: Resistance and Habitat calculator tool (McRae et al., 2013). Then, the resulting habitat layer was used to obtain the collection of suitable habitat patches with the Core Area Mapper toolkit in the Gnarly Landscape Utilities toolbox (Shirk & McRae, 2013), with an assigned minimum habitat threshold per pixel of 0.5 and a moving window equal to the size of a single cell. The least‐cost distance among the core areas (input ii) was calculated using Graphab 2.4 (Foltête et al., 2012). A complete graph, which calculates all the connections among nodes, was calculated with a cost threshold of 100,000 and a probability of dispersion among patches of 0.5 when the dispersion distance was 1 km.

## 3 RESULTS

The test for Hardy–Weinberg equilibrium showed that all loci but one (‐OM07) showed significant deviations from equilibrium in at least one population. However, deviations were not consistently observed in the same loci across populations (Table S3). The analysis in Micro‐Checker suggested the presence of null alleles at all the loci except for quru‐GAOC11 and ‐0E09. Four loci (‐IF02, ‐2F05, IF07, and ‐OM07) showed a null allele frequency below 8%, and ‐OI01, ‐IC08, ‐OC19, and ‐OA01 showed null allele frequencies of 0.10, 0.11, 0.21, and 0.22, respectively. The estimations performed separately by sampling location using FreeNa showed, in general, low null allele frequencies (Table S4).

The results for the simultaneous calculation of inbreeding coefficients and null allele frequencies by INEST showed that the model with the lowest DIC included the three possible parameters (null alleles [n], inbreeding [f], and genotyping failures [b]; DIC = 14,859.53). The average inbreeding coefficient was 0.0875 (0.0384, 0.1258), and the null allele frequency was lower than 0.2 for all loci (Table S5). The estimation of $F_{\mathrm{ST}}$ with and without adjustment for null alleles showed very similar values (0.066 [95% CI = 0.039–0.098] and 0.064 [95% CI = 0.039–0.093], respectively). Therefore, we concluded that the influence of null alleles on the estimation of genetic structure was low and decided to continue the analysis using all the analyzed loci. Finally, the linkage disequilibrium test detected significant linkage among every pair of loci in at least one sampling location. However, there was no consistent pattern across the sampling locations.

### 3.1 Genetic diversity

**FIGURE 4:**
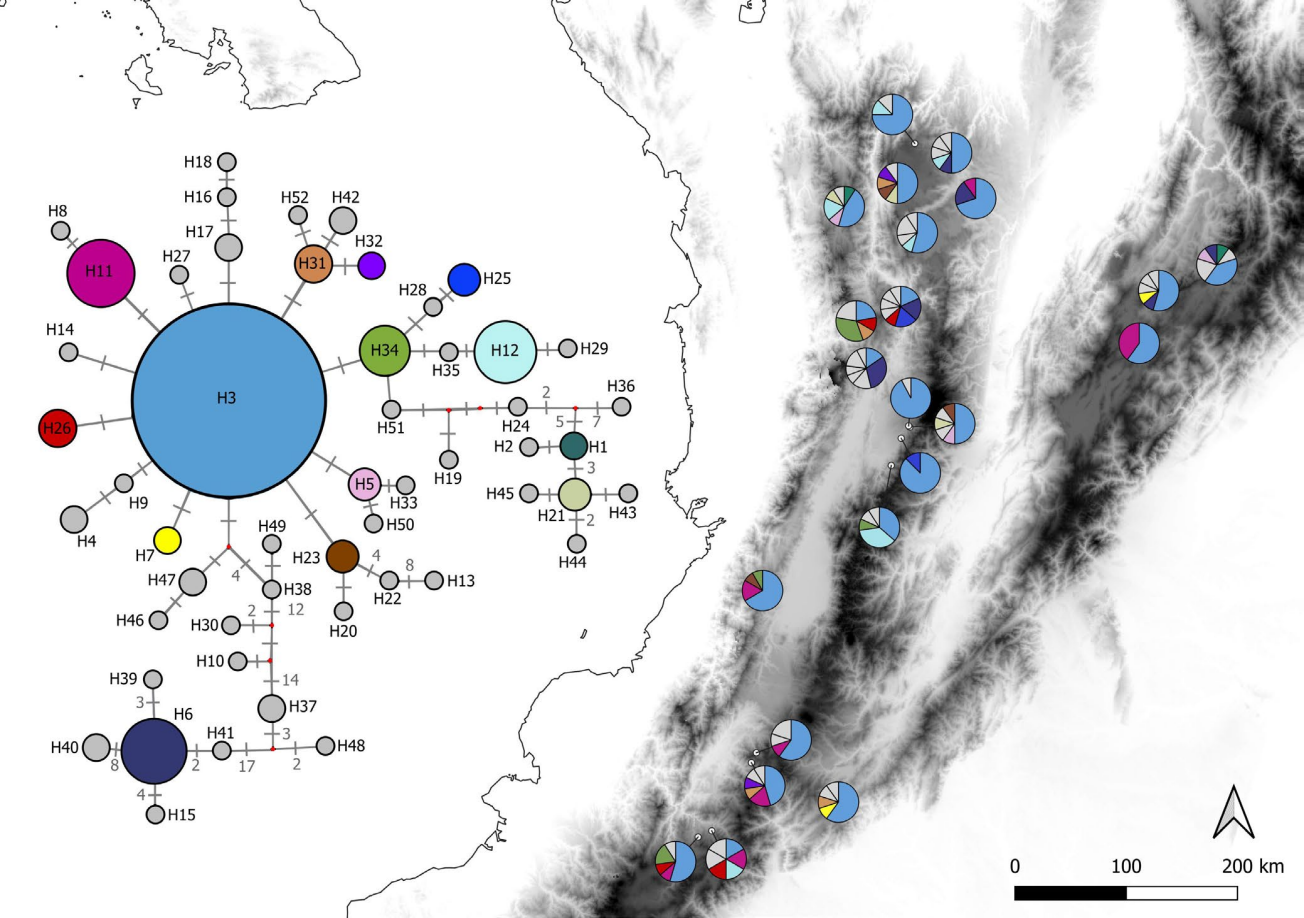
Distribution of 52 haplotypes identified from cpSSR in sampling locations (pie charts) and haplotype network inferred using neighbor joining in *Q. humboldtii*. Gray color represents haplotypes that occur in a single sampling location. Single mutational steps are indicated by a gray bar perpendicular to the network linkages, when the number of mutational is higher than one it is indicated by a gray number. Elevation of the Colombian Andes is represented in a grayscale from lowlands (white) to highlands (black)

For the 22 sampling locations evaluated, 52 different cpSSR haplotypes with 38 unique haplotypes (present in a single population) and three widely distributed haplotypes were identified (Figure 4). The number of haplotypes per population ranged from two to eight (Table S6). At the regional level, 14 (8 unique) haplotypes were observed on Cordillera Oriental, 32 (21 unique) on Cordillera Central and 23 (11 unique) on Cordillera Occidental. The within‐population genetic diversity (h) varied between 0.154 (sampling location nine; Table S6) and 1 (sampling location 21; Table S6), with mean h (*SE*) and *v* (*SE*) values of 0.69 (0.209) and 1.74 (1.25), respectively.

A mean allelic richness per population of 6.095 (0.047) and a mean effective allelic richness of 3.93 (0.0343) were found for the nuclear microsatellites (Table S6). The number of private alleles per population ranged from zero to five. Regionally, the mean allelic richness was 15.6 (36 private) for Cordillera Central, 10.6 (14 private) for Cordillera Oriental, and 12.2 (10 private) for Cordillera Occidental. The mean expected heterozygosity was 0.737 (0.002), and the mean observed heterozygosity was 0.647 (0.005) (Table S6).

### 3.2 Genetic structure

The AMOVA for the cpSSR haplotypes suggested that considering both the infinite allele mutation model (IAM) and the stepwise mutation model (SMM), most of the genetic variation was distributed within the sampling locations (81.11% for the IAM and 78.7% for the SMM). The fixation indices had low values ($F_{\mathrm{ST}}$ = 0.18, *p* < .001; $R_{\mathrm{ST}}$ = 0.21, *p* < .0001), which indicated low‐to‐moderate levels of differentiation among the sampling locations. Moreover, genetic differentiation among regions (Cordillera Occidental, Cordillera Central and Cordillera Oriental) was not supported by the AMOVA results under either mutation model (IAM 3.4%, *p*  = .178 and SMM 2.25%, *p* = .29, respectively).

Similar to the chloroplast results, the AMOVA calculation for nSSR using the IAM showed that most of the genetic variation was distributed within the sampling locations (92.82%), and a very low $F_{\mathrm{ST}}$ value was estimated (0.07, *p* < .0001). However, the results obtained using the SMM indicated a higher percentage of variation among the sampling locations within regions (56.71%; $R_{\mathrm{ST}}$ = 0.55, *p* < .0001). With regard to the comparisons among regions, the nuclear microsatellites showed no variation for either mutation model.

**FIGURE 5:**
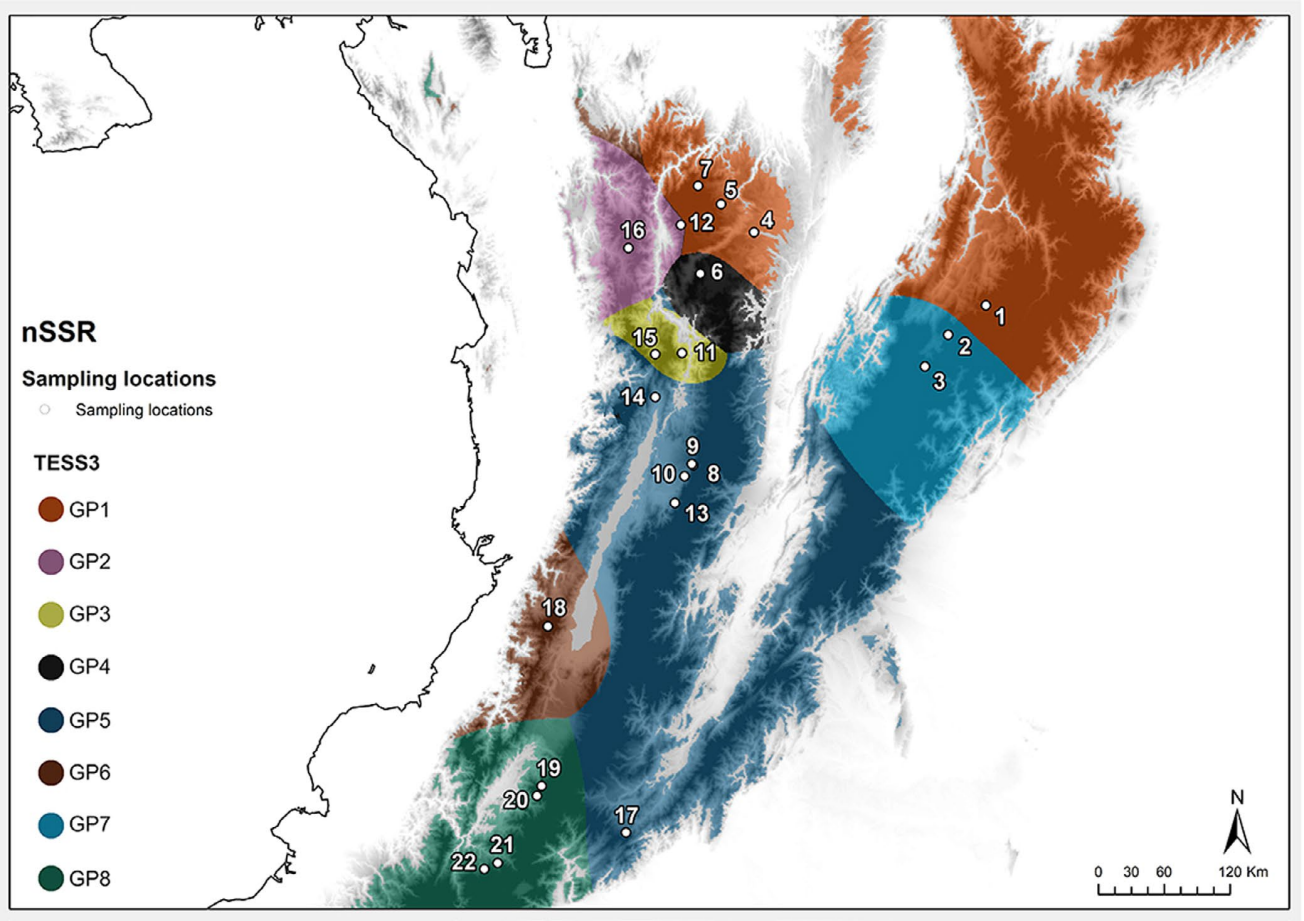
Distribution of genetic clusters calculated by tess3r (background coloration) from nSSR. Elevation is represented by a grayscale of light colors for lowlands and dark colors for highlands

In the analysis of the genetic structure accounting for geographic distance, the lowest median nSSR value for the cross‐validation criterion had an RMSE = 0.198, indicating the presence of eleven clusters. The interpolated results showed only eight groups due to the low ancestry proportion represented in the remaining genetic groups (Figure 5), which are described as follows. The Cordillera Occidental contained four clusters (GP2, GP3, GP5, and GP6), three of which were solely restricted to this region (GP2, GP3, and GP6). Similarly, the Cordillera Central was represented by four clusters (GP1, GP4, GP5, and GP6), with GP4 being the only restricted cluster. Finally, Cordillera Oriental contained three clusters (GP1, GP7, and GP5), of which GP5 was unique to this region.

### 3.3 Phylogeographic structure

The genetic differentiation among the sampling locations for the ordered alleles ($N_{\mathrm{ST}}$) was 0.168 and the $pN_{\mathrm{ST}}$ was 0.0569 (0.0132). The observed value was higher than expected under random permutations (*p* = .000), suggesting the presence of phylogeographic structure based on the cpSSR loci. The allele size permutation test ($R_{\mathrm{ST}}$ vs. $pR_{\mathrm{ST}}$) resulted in a significantly larger value of $R_{\mathrm{ST}}$ (0.53) than of $pR_{\mathrm{ST}}$ (0.11) (*SD* 0.04; *p* = .000), also indicating the presence of phylogeographic structure in the nSSR loci. The calculated haplotype network showed 52 haplotypes, which were separated in most of the cases by a single mutational step (Figure 4). The haplotypes were characterized by low frequencies and restricted distributions, except for H3, which was present in all the sampling locations and had the highest frequency, followed by H11, H12, and H6, which were recorded at 32%, 27%, and 27% of the sampling locations, respectively (Figure 4). The haplotype network showed that most of the unique haplotypes were randomly distributed throughout the Andean oak sampling locations. Only population 14, which is located at the border between Cordillera Oriental and the Magdalena Valley, showed a high number of unique haplotypes (five haplotypes) (Figure 4).

### 3.4 Historical population demography

The ABC analyses showed that scenario three was the best model for explaining *Q. humboldtii* demographic changes, according to the direct estimates (1.0, 95% CI: 1.0–1.0) and logistic regression tests (1.0, 95% CI: 1.0–1.0) (Table 1). The posterior error rate was 0.000 for both logistic and direct approaches. The type I error rate, meaning the proportion of times scenario three was rejected even though it was true, was 0.099. The overall type II error rate, meaning the proportion of times scenario three had the highest posterior probability even though it was false, was 0.008 and 0.0135 for direct and logistic approaches (Table 1). Scenario three tested the hypothesis of a bottleneck event followed by a recent demographic expansion. The average posterior distribution suggested that the species suffered the bottleneck event 47,500 years BP (q05: 24,000 years; q95: 67,700 years) and a subsequent demographic expansion approximately 28,400 years ago (q05: 9,690 years; q95: 46,100 years) (Table S7; Figure 3b).

**TABLE 1:** DIYABC results for model comparison

Scenarios	Posterior probability	Confidence intervals (0.05–0.95)	Direct *p*(Scenario X |Scenario 3) (logistic)	Direct *p*(Scenario 3| Scenario X) (logistic)
Scenario 1: Constant population size	0.000	0.000–0.000	0.051 (0.038)	6 (9)
Scenario 2: Constant expansion	0.000	0.000–0.000	0.048 (0.043)	11 (18)
Scenario 3: Bottle neck and expansion	1.000	1.000–1.000	0.901 (0.019)	–
			**Direct type I error** **(logistic)**	**Direct type 2 error** **(logistic)**
			0.099 (0.081)	0.0085 (0.0135)

Note

Logistic regression posterior probability and 95% confidence intervals (CI) of three demographic scenarios. In the last two columns, it is shown the probability of scenarios one, two, or three of having the highest posterior probability given that scenario three was true (*p*(Scenario X| Scenario 3)); and the probability of scenario three of having the highest posterior probability even though it was false (*p*(Scenario 3| Scenario X)). In the inferior part of the table, type I and type II error rates are estimated for scenario 3.

### 3.5 Connectivity

**FIGURE 6:**
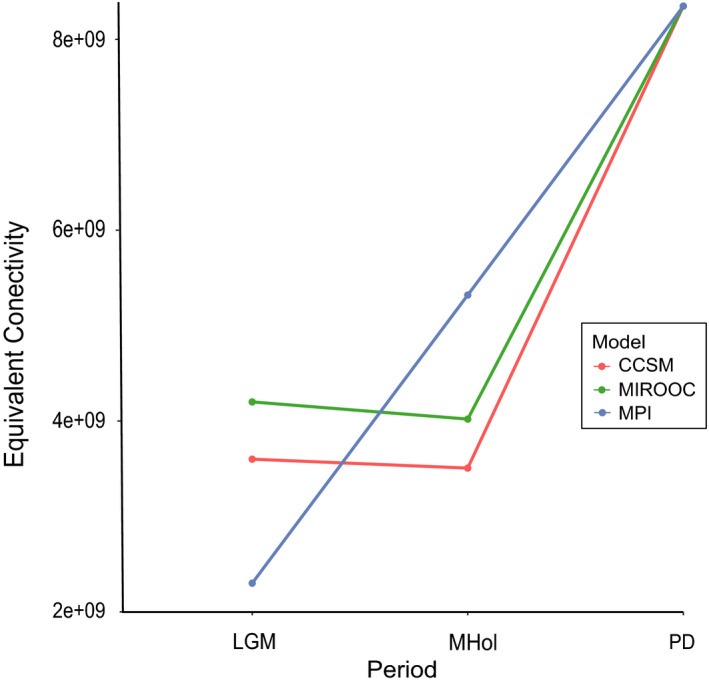
Equivalent connectivity area (ECA) in m^2^ for three periods: LGM (~21 ka), MHol (~11 ka), and PD (present day) and three global climate models (CCSM4, MIROC‐ESM, and MPI). This connectivity index calculates the area of a single patch with maximum connectivity that would reflect the same connectivity value as the observed landscape

Our estimations of habitat connectivity among the three time periods (LGM, MHol, and PD) showed an increase in the ECA from the past to the present (Figure 6). There were varying results depending on the model used for the calculations. The results based on the CCSM and MIROC models were similar, in contrast with the results based on the MPI model. During the LGM, the lowest ECA value was obtained with the MPI model (2,300 km^2^), followed by the CCSM (3,600 km^2^) and MIROC (4,200 km^2^) models. During MHol, the CCSM and the MIROC models resulted in a slight decrease in connectivity (3,506 km^2^ and 4,020 km^2^, respectively), while the MPI model predicted a substantial increase (5,320 km^2^), and the ECA for the present time was 8,348 km^2^.

## 4 DISCUSSION

In recent years, complex phylogeographic patterns have been described for Neotropical species (Gutiérrez‐García & Vázquez‐Domínguez, 2013; Ornelas et al., 2013), and both climate and topography have arisen as major drivers of species evolution (Mastretta‐Yanes et al., 2015; Ramírez‐Barahona & Eguiarte, 2013). Unfortunately, few studies have aimed to describe the effects of the interplay between topography and historical climate changes on the biological communities in most of the montane ecosystems of the northern Andes, such as montane and submontane forests. Therefore, our study was directed at increasing the understanding of the population history of trees species from this area. To do so, we developed analyses of genetic diversity, phylogeographic structure, and historical demography and compared the results to the topographical and paleoclimatic history of the region for the widely distributed *Q. humboldtii,* a lonely oak species that has reached and successfully colonized the Colombian Andes.

### 4.1 *Quercus humboldtii* genetic diversity

The nuclear genetic diversity of *Q. humboldtii* in the Colombian Andes fell within the range of values observed in other oak species (Appendix S2 for methods and Table S8). Surprisingly, our results differed from previous genetic studies on *Q. humboldtii* (Fernández‐M & Sork, 2005, 2007), although the comparison should be taken with caution since there is a large difference in the number of loci and the spatial scale with respect to our study. In contrast with the findings of the nuclear diversity, *Q. humboldtii's* chloroplast genetic diversity was higher than the values found in the oak species of Europe and North America, but was variable with regard to the values found in oak species in Mexico, Central America, and Asia (Table S9).

It has been previously proposed that the high genetic diversity that characterizes several oak species can be explained by intrinsic life‐history traits that allow large effective populations to be maintained over time (Kremer & Hipp, 2019). We observed high nuclear genetic diversity, suggesting that *Q. humboldtii* was able to sustain sufficiently large populations and accumulate genetic diversity through the climatic oscillations of the Pleistocene. Furthermore, it suggests that a relatively more stable climate may have allowed the accumulation of genetic variation in contrast with the more difficult conditions that temperate species had to endure during glacial periods.

### 4.2 *Quercus humboldtii* genetic structure

Genetic structure analyses (AMOVA and *tess3r*) indicated a low genetic structure. Strong physiographic features, such as the Cauca and Magdalena valleys, which descend by up to 100 and 900 m, respectively (Josse et al., 2009), did not segregate the sampling locations into different genetic groups, probably indicating historical connections throughout these valleys. Therefore, despite the altitudinal changes in the vegetation belts and the possible fragmentation of patches, our data suggested that connectivity among Andean forest patches was maintained throughout the climatic variations in the late Pleistocene.

It is difficult to compare our results with those of previous studies in the region due to the few phylogeographic analyses of mid‐elevation species similar to *Q. humboldtii*. However, results obtained for the palm genus *Ceroxylon* (Sanín et al., 2017), a group that shares several characteristics with *Q. humboldtii*, such as altitudinal distribution, life span, and aggregation pattern, showed partial congruence with our findings. The authors determined that the populations in the center of the distribution of the three species from the northern Andes (*C. quindiense*, *C. ventricosum,* and *C. ceriferum*) have had higher immigration rates and maintained higher demographic stability than the peripheral populations. Interestingly, they proposed that the persistence of these populations is related to the relatively stable temperature and humidity conditions in Colombia during the last 350 ka.

Sanín et al. (2017) mentioned that even during glacial periods, it is likely that the altitudinal descent of the vegetation belt did not compromise the persistence and connectivity of *Ceroxylon* populations in the northern Andes. A similar scenario of persistent connectivity throughout its geographic range may also explain the lack of genetic structure observed in *Q. humboldtii*. However, it is worth mentioning that important differences in the ecology of these palm species, such as climatic specificity, endemism, and relatively short pollination and dispersal distances, have resulted in different patterns of genetic structure than those found in *Q. humboldtii*.

### 4.3 *Quercus humboldtii* responses to climatic fluctuations during the Pleistocene

There are three indications that *Q. humboldtii* went through a bottleneck in the recent past: the higher expected than observed heterozygosity of the nuclear microsatellites, the star‐shaped haplotype network observed in the chloroplast data, and the ABC demographic scenario of a contraction of the population (47.5 kyr BP) followed by a demographic population expansion (28.4 kyr BP) just before the LGM (~21 kyr BP) (Figure 3b). Interestingly, the demographic fluctuations inferred from our molecular data are congruent with equivalent fluctuations in the abundance of *Quercus* in the fossil record that are dated to the beginning of the last glacial period (44 ka BP). This epoch has been described as climatically cool and wet, although characterized by several stadial and interstadial fluctuations that caused variation in the extension of the glaciers and the altitudinal position of vegetation belts (van der Hammen & Cleef, 1986; van der Hammen et al., 1973; Hooghiemstra, 2006). Several climatic fluctuations occurred through the end of the Pleistocene and the Holocene, although none of them seem to have left a genetic imprint detectable using microsatellite markers.

Alternatively, demographic fluctuations could be related to biotic interactions. Throughout the phases with abundant forests during these periods, *Quercus* became a common element of the Andean forest (van der Hammen et al., 1973). Nonetheless, from 58 to 35 ka BP, there was a change in the forest composition, and *Polylepis*, a genus usually considered to be in competition with *Quercus*, increased its contribution to the pollen record (up to 60%), while *Quercus* gradually decreased (down to 20%) (Hooghiemstra, 2006). At the highest peak in *Polylepis* representation, the upper forest limit occurred at 2,600–2,800 m, which indicates the prevalence of cold climatic conditions (van der Hammen & Cleef, 1986; Hooghiemstra, 2006). There is no clear explanation for this change in composition, although it is hypothesized that it was not driven by temperature changes (Hooghiemstra, 2006). Therefore, it is possible that both the observed fluctuation in the pollen record abundance and the demographic changes could have resulted not only from climatic fluctuations but also from competition between *Quercus* and other dominant genera in high‐altitude forest belts.

### 4.4 *Quercus humboldtii* historical connectivity

Our results suggest an increase in connectivity (ECA) and an altitudinal displacement of the highly suitable areas for *Q. humboldtii* from the past to the present (see the results and description of the ENM results in Figure  S1and Appendix S3). A similar pattern was found in the ECA of the available surface area for the altitudinal ranges suggested by paleoecological evidence for Andean (UMF) and sub‐Andean (LMF) forests during the LGM and the present (Flantua & Hooghiemstra, 2017; Hooghiemstra & Flantua, 2019). This suggests that the change in connectivity for areas with environmentally suitable conditions for *Q. humboldtii* may be explained by the interaction between the topography of the distributional ranges it occupied and the climatic oscillations that occurred during the late Pleistocene.

A similar conclusion has been reached in the Hengduan Mountains in China, where range expansions have been reported in subalpine and mid‐elevation species from the LGM to the present (Liang et al., 2018). These authors explained such range expansions as a result of the increase in the available surface area during altitudinal shifts from cold to warm periods in a landscape with complex topography. The results from Liang et al. (2018) together with our results add to the evidence supporting the prediction made by Elsen and Tingley (2015), which states that contrary to the common expectation of decreasing superficial area as elevation increases, some hypsographic patterns of mountainous landscapes may result in range expansions for upslope species range shifts.

On the other hand, a few evolutionary models have been proposed for mountain species in the Neotropics with specific genetic expectations that can be tested with phylogeographic data (Flantua et al., 2019; Mastretta‐Yanes et al., 2015; Ramírez‐Barahona & Eguiarte, 2013). These models have been proposed for island‐like ecosystems (cloud forests and alpine ecosystems). Therefore, we do not expect the genetic patterns of *Q. humboldtii*, a species with a wide altitudinal distribution that is capable of living in different environmental conditions, to coincide with the proposed genetic predictions. However, the most recent models (Flantua et al., 2019; Mastretta‐Yanes et al., 2015) indicate that genetic differentiation and historical connectivity (respectively) are functions of topography. We sustain that the same idea can be applied to understand the evolutionary history of mid‐elevation forests. Our results suggest that the North Andean topography at the altitudinal ranges occupied by *Q. humboldtii* allowed continuous connectivity among forest patches through recent times.

It has been previously proposed that distinct phylogeographic patterns might be shown by species at different elevations (Kropf et al., 2003). Therefore, to further test whether the results shown here are a common pattern among mid‐elevation species, we suggest that genetic data should be tested against geographically explicit predictions, that is, by calculating the amount of available surface area and the connectivity among the altitudinal bands encompassed by a species’ range.

## 5 CONCLUSIONS

Our study on the phylogeography of *Q. humboldtii* contributes to the understanding of the effect of climatic fluctuations in the late Pleistocene on the evolution of species found in Andean mountain forests. We found a high population genetic diversity and lack of genetic structure, which were explained by the increase in historical connectivity, supporting the prediction of Elsen and Tingley (2015) about the effect of different topographical contexts for shifts in altitudinal ranges. Furthermore, there is evidence of a recent demographic expansion that predates the LGM, which may reflect a change in the dominance among floristic elements of the Andean forests, as has been reported in paleoecological studies for the region (Hooghiemstra, 2006).

The scarcity of phylogeographic studies for this region, particularly for the northern Andes, highlights the urgent need for further work to compare the phylogeographic response of different groups of Andean and sub‐Andean plants. Such studies are crucial for better understanding the general evolutionary processes that caused the current distribution of genetic variation and species ranges in this region. Finally, a general understanding of the vegetation response through past climatic fluctuations is important for guiding future research and management actions under present‐day conservation challenges, such as climate change, habitat loss, and fragmentation. Further studies can evaluate the genetic consequences, such as a decrease in gene flow, an increase in inbreeding, and a reduction in genetic diversity, that these factors can have with knowledge of the historical processes that have shaped the species in this region.

## CONFLICT OF INTEREST

The authors have no competing interests to declare.

## AUTHOR CONTRIBUTIONS

**Sofía Zorrilla‐Azcué:** Data curation (equal); formal analysis (equal); investigation (equal); methodology (equal); writing‐original draft (equal); writing‐review & editing (equal). **Antonio González‐Rodríguez:** Conceptualization (equal); funding acquisition (equal); investigation (equal); methodology (equal); writing‐review & editing (equal). **Ken Oyama:** Conceptualization (equal); funding acquisition (equal); investigation (equal); methodology (equal); writing‐review & editing (equal). **Mailyn A. González:** Data curation (equal); funding acquisition (equal); investigation (equal); writing‐review & editing (equal). **Hernando Rodríguez‐Correa:** Conceptualization (equal); formal analysis (equal); funding acquisition (equal); investigation (equal); methodology (equal); writing‐original draft (equal); writing‐review & editing (equal).

## Supporting information

Fig S1

Supplementary Material

## Data Availability

All genetic and geographic data are available in Dryad (https://doi.org/10.5061/dryad.08kprr528).
