# Supplementary figures and images for: The DNA history of a lonely oak: Quercus humboldtii phylogeography in the Colombian Andes
Source: Ecol Evol. 2021 May 7;11(11):6814–28. doi: 10.1002/ece3.7529 (PMC8207385; doi:10.1002/ece3.7529)

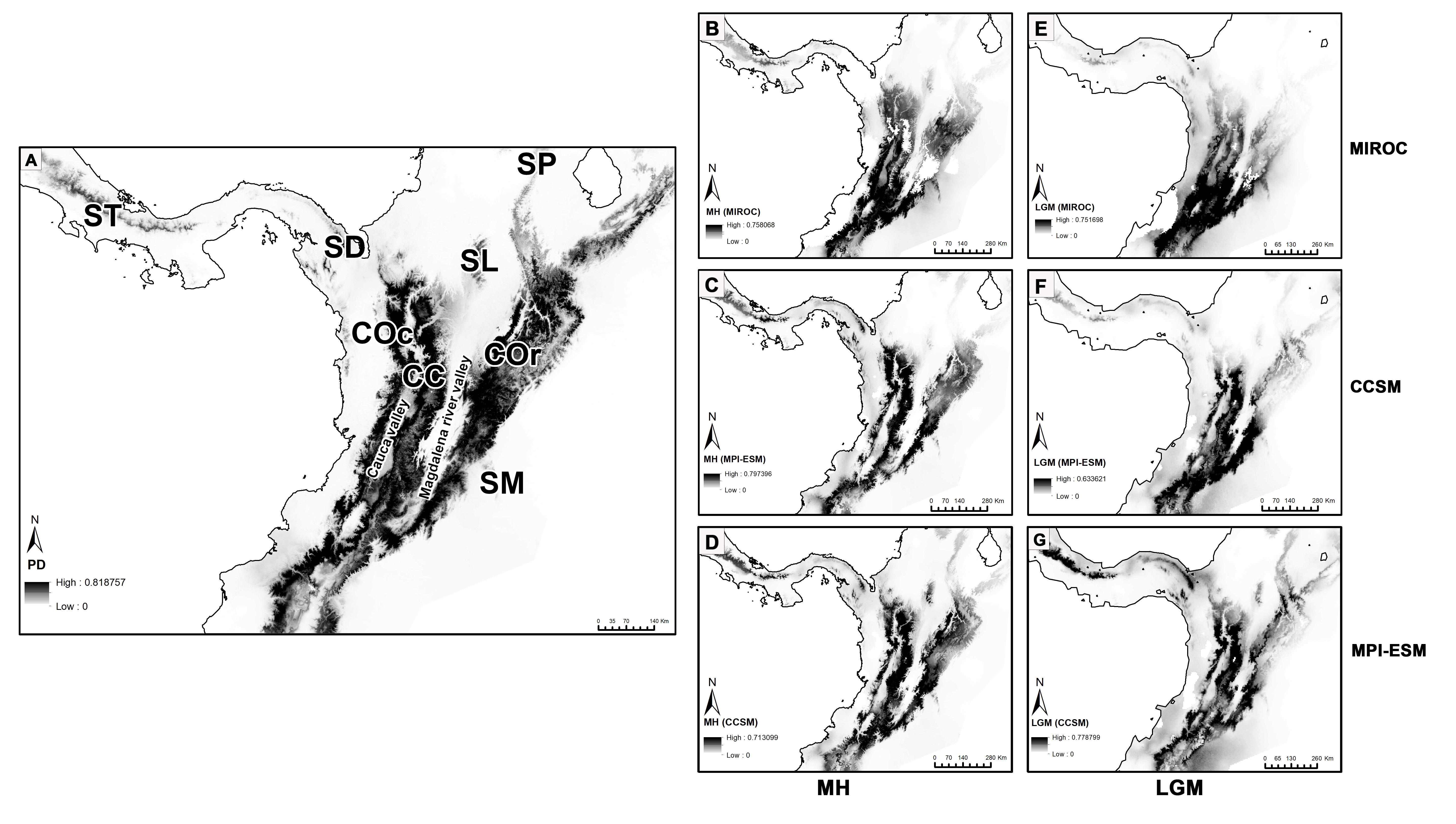

Supplement: Supplementary file 1 — Fig S1 [file ECE3-11-6814-s002.jpg]
