# Supplementary material for: The DNA history of a lonely oak: Quercus humboldtii phylogeography in the Colombian Andes
Source: Ecol Evol. 2021 May 7;11(11):6814–28. doi: 10.1002/ece3.7529 (PMC8207385; doi:10.1002/ece3.7529)
Supplement: Supplementary file 2 — Supplementary Material [file ECE3-11-6814-s001.docx]

# Appendix S1

## Table S1. Population geographic information summary. COr: Cordillera Oriental; CC: Cordillera Central; COc: Cordillera Occidental; $\boldsymbol{n}$: sample size (for nuclear and chloroplast microsatellites).

| **ID** | **Region** | **Latitude** | **Longitude** | **Elevation (m.a.s.l.)** | **n**  **(nSSR)** | **n**  **(cpSSR)** |
| --- | --- | --- | --- | --- | --- | --- |
| 1 | COr | 5.950 | -73.150 | 3,031 | 10 | 10 |
| 2 | COr | 5.710 | -73.460 | 2,781 | 10 | 11 |
| 3 | COr | 5.450 | -73.650 | 2,988 | 10 | 10 |
| 4 | CC | 6.550 | -75.050 | 9,73 | 9 | 10 |
| 5 | CC | 6.780 | -75.320 | 2,017 | 9 | 10 |
| 6 | CC | 6.210 | -75.490 | 2,475 | 11 | 11 |
| 7 | CC | 6.930 | -75.510 | 2,701 | 8 | 8 |
| 8 | CC | 4.640 | -75.550 | 1,965 | 10 | 10 |
| 9 | CC | 4.650 | -75.560 | 1,965 | 13 | 13 |
| 10 | CC | 4.550 | -75.620 | 1,900 | 8 | 8 |
| 11 | COc | 5.560 | -75.640 | 2,242 | 9 | 11 |
| 12 | CC | 6.610 | -75.650 | 2,923 | 10 | 10 |
| 13 | CC | 4.330 | -75.700 | 2,052 | 9 | 11 |
| 14 | COc | 5.200 | -75.860 | 1,499 | 11 | 13 |
| 15 | COc | 5.550 | -75.860 | 1,994 | 9 | 9 |
| 16 | COc | 6.420 | -76.080 | 2,889 | 10 | 11 |
| 17 | COr | 1.630 | -76.100 | 1,716 | 10 | 10 |
| 18 | COc | 3.320 | -76.740 | 1,801 | 11 | 12 |
| 19 | CC | 2.010 | -76.790 | 2,446 | 10 | 10 |
| 20 | CC | 1.930 | -76.830 | 2,591 | 9 | 11 |
| 21 | CC | 1.380 | -77.150 | 1,790 | 5 | 6 |
| 22 | CC | 1.330 | -77.260 | 2,314 | 10 | 11 |

## Table S2. DIYABC prior range values for all scenarios (see Figure 3 A for graphical representation of scenarios). Effective population sizes are represented by N, the first subscript represents order in time and the second represents the scenario in which the parameter was used (i.e. N_11,2_ is the parameter for most recent population sizes shared in scenarios 1 and 2). Time (number of generations) was represented as t with higher values corresponding to events further in the past. Additional restrictions were imposed as follows: t_2_>t_1_, N_22_> N_11,2_, N_32_< N_22_, N_32_< N_11,2_, N_23_< N_13_

| **Parameter** | **Prior distribution** | **Minimum** | **Maximum** |
| --- | --- | --- | --- |
| t_1_ | Uniform | 10 | 500 |
| t_2_ | Uniform | 10 | 700 |
| N_11,2_ | Uniform | 40000 | 100000 |
| N_22_ | Uniform | 10 | 100000 |
| N_32_ | Uniform | 10 | 100000 |
| N_13_ | Uniform | 40000 | 100000 |
| N_23_ | Uniform | 10 | 700 |
| N_33_ | Uniform | 40000 | 100000 |

## Table S3. Results from Hardy-Weinberg equilibrium test for nSSR loci, showing the number of populations with significant deviations from equilibrium at the corresponding loci.

| **Locus** | **Number of populations showing significant HWE deviations** |
| --- | --- |
| 2F05 | 4 |
| IC08 | 9 |
| IF02 | 3 |
| IF07 | 2 |
| OA01 | 14 |
| OC11 | 0 |
| OC19 | 9 |
| OE09 | 1 |
| OI01 | 1 |
| OM07 | 1 |

## Table S4. Null allele frequencies by sampling location and locus estimated by FreeNA.

| **Sampling**  **location** | **IF07** | **IC08** | **OM07** | **OE09** | **2F05** | **IF02** | **OC11** | **OA01** | **OI01** | **OC19** |
| --- | --- | --- | --- | --- | --- | --- | --- | --- | --- | --- |
| 1 | 0.064 | 0.067 | 0.066 | 0.000 | 0.000 | 0.000 | 0.000 | 0.000 | 0.091 | 0.024 |
| 2 | 0.130 | 0.251 | 0.000 | 0.000 | 0.031 | 0.028 | 0.000 | 0.000 | 0.000 | 0.000 |
| 3 | 0.000 | 0.233 | 0.000 | 0.000 | 0.000 | 0.000 | 0.033 | 0.000 | 0.000 | 0.245 |
| 4 | 0.031 | 0.187 | 0.000 | 0.077 | 0.057 | 0.000 | 0.000 | 0.355 | 0.074 | 0.233 |
| 5 | 0.011 | 0.000 | 0.026 | 0.031 | 0.000 | 0.000 | 0.000 | 0.123 | 0.037 | 0.176 |
| 6 | 0.000 | 0.119 | 0.000 | 0.000 | 0.101 | 0.000 | 0.112 | 0.122 | 0.000 | 0.000 |
| 7 | 0.042 | 0.000 | 0.263 | 0.000 | 0.015 | 0.000 | 0.000 | 0.000 | 0.056 | 0.000 |
| 8 | 0.000 | 0.000 | 0.000 | 0.000 | 0.000 | 0.000 | 0.000 | 0.185 | 0.125 | 0.166 |
| 9 | 0.084 | 0.034 | 0.000 | 0.057 | 0.084 | 0.095 | 0.119 | 0.310 | 0.085 | 0.273 |
| 10 | 0.000 | 0.045 | 0.000 | 0.000 | 0.000 | 0.205 | 0.000 | 0.330 | 0.108 | 0.178 |
| 11 | 0.001 | 0.039 | 0.000 | 0.000 | 0.000 | 0.000 | 0.000 | 0.130 | 0.000 | 0.080 |
| 12 | 0.000 | 0.000 | 0.000 | 0.000 | 0.000 | 0.000 | 0.000 | 0.095 | 0.000 | 0.310 |
| 13 | 0.000 | 0.179 | 0.000 | 0.000 | 0.056 | 0.000 | 0.000 | 0.267 | 0.000 | 0.287 |
| 14 | 0.000 | 0.000 | 0.030 | 0.157 | 0.086 | 0.061 | 0.103 | 0.120 | 0.141 | 0.279 |
| 15 | 0.000 | 0.000 | 0.000 | 0.000 | 0.000 | 0.030 | 0.070 | 0.199 | 0.354 | 0.207 |
| 16 | 0.086 | 0.229 | 0.000 | 0.091 | 0.000 | 0.000 | 0.000 | 0.092 | 0.004 | 0.000 |
| 17 | 0.078 | 0.000 | 0.000 | 0.101 | 0.000 | 0.025 | 0.000 | 0.098 | 0.183 | 0.102 |
| 18 | 0.000 | 0.237 | 0.000 | 0.000 | 0.034 | 0.031 | 0.000 | 0.033 | 0.000 | 0.033 |
| 19 | 0.000 | 0.000 | 0.045 | 0.000 | 0.105 | 0.042 | 0.000 | 0.276 | 0.021 | 0.197 |
| 20 | 0.111 | 0.059 | 0.074 | 0.000 | 0.112 | 0.000 | 0.066 | 0.184 | 0.146 | 0.258 |
| 21 | 0.000 | 0.000 | 0.000 | 0.000 | 0.000 | 0.000 | 0.000 | 0.001 | 0.172 | 0.122 |
| 22 | 0.064 | 0.000 | 0.000 | 0.000 | 0.000 | 0.000 | 0.000 | 0.000 | 0.004 | 0.438 |
| **Mean** | 0.032 | 0.076 | 0.023 | 0.023 | 0.031 | 0.023 | 0.023 | 0.133 | 0.073 | 0.164 |
| **s.d.** | 0.042 | 0.095 | 0.058 | 0.044 | 0.041 | 0.047 | 0.041 | 0.117 | 0.088 | 0.122 |

## Table S5. Results of null allele frequencies from the model nfb calculated for nSSR loci with the program INEST for all individuals

| **Locus** | **Mean** | **Q(5.0%)** | **Q(95.0%)** |
| --- | --- | --- | --- |
| IF07 | 0.015 | 0 | 0.045 |
| IC08 | 0.047 | 0.004 | 0.091 |
| OM07 | 0.012 | 0 | 0.038 |
| 0E09 | 0.004 | 0 | 0.017 |
| 2F05 | 0.010 | 0 | 0.036 |
| IF02 | 0.012 | 0 | 0.042 |
| OC11 | 0.007 | 0 | 0.026 |
| OA01 | 0.180 | 0.135 | 0.230 |
| OI01 | 0.058 | 0.013 | 0.103 |
| OC19 | 0.151 | 0.108 | 0.195 |

## Table S6. Genetic diversity estimators for both molecular markers (cpSSR and nSSR). ID from Table S1; Rarefied haplotype richness (k = 6); $\boldsymbol{h}$: genetic diversity for unordered alleles; $\boldsymbol{v}$: gene diversity with ordered alleles; A: number of alleles; $\boldsymbol{A}_{\boldsymbol{e}}$: number of effective alleles; $\boldsymbol{A}_{\boldsymbol{p}}$: number of private alleles; $\boldsymbol{H}_{\boldsymbol{o}}$: observed heterozygosity; $\boldsymbol{uH}_{\boldsymbol{e}}$: unbiased expected heterozygosity, AR: rarefied allelic richness.

|  | **cpSSR** | | | | **nSSR** | | | | | |
| --- | --- | --- | --- | --- | --- | --- | --- | --- | --- | --- |
| ID | Haplotype  richness | Rarefied haplotype richness | *h* | *v* | *A* | $A_{e}$ | $A_{p}$ | $H_{o}$ | *u*$H_{e}$ | *AR* |
| 1 | 6 | 3.78 | 0.844 | 2.6 | 6.700 | 4.108 | 0 | 0.730 | 0.768 | 5.47 |
| 2 | 6 | 4.26 | 0.727 | 2.909 | 6.300 | 4.250 | 1 | 0.720 | 0.766 | 5.26 |
| 3 | 2 | 3.73 | 0.533 | 0.533 | 6.600 | 4.381 | 2 | 0.740 | 0.757 | 5.49 |
| 4 | 3 | 2.00 | 0.511 | 3 | 6.000 | 3.746 | 1 | 0.533 | 0.740 | 4.84 |
| 5 | 6 | 2.47 | 0.778 | 3.511 | 6.100 | 3.490 | 1 | 0.700 | 0.736 | 4.85 |
| 6 | 5 | 4.00 | 0.709 | 1.164 | 6.400 | 4.303 | 0 | 0.709 | 0.759 | 5.35 |
| 7 | 3 | 3.45 | 0.464 | 0.929 | 5.300 | 3.510 | 0 | 0.707 | 0.733 | 4.50 |
| 8 | 6 | 2.50 | 0.778 | 1.111 | 6.200 | 3.920 | 5 | 0.670 | 0.742 | 5.19 |
| 9 | 2 | 4.00 | 0.154 | 0.154 | 6.200 | 4.037 | 3 | 0.531 | 0.757 | 5.36 |
| 10 | 2 | 1.46 | 0.250 | 0.25 | 5.900 | 4.308 | 1 | 0.637 | 0.770 | 4.88 |
| 11 | 8 | 1.75 | 0.946 | 3.964 | 6.000 | 3.932 | 2 | 0.700 | 0.762 | 5.04 |
| 12 | 6 | 5.18 | 0.778 | 1.178 | 6.800 | 4.530 | 5 | 0.730 | 0.763 | 5.66 |
| 13 | 5 | 4.00 | 0.782 | 1.418 | 4.900 | 2.777 | 2 | 0.524 | 0.622 | 4.11 |
| 14 | 7 | 3.61 | 0.885 | 4.885 | 6.200 | 3.629 | 3 | 0.527 | 0.705 | 5.01 |
| 15 | 5 | 4.53 | 0.861 | 1.528 | 5.000 | 3.461 | 1 | 0.578 | 0.722 | 4.38 |
| 16 | 6 | 4.15 | 0.800 | 1.164 | 6.300 | 4.209 | 4 | 0.680 | 0.754 | 5.14 |
| 17 | 5 | 4.00 | 0.667 | 1.111 | 6.200 | 3.254 | 0 | 0.590 | 0.689 | 4.92 |
| 18 | 4 | 3.40 | 0.561 | 0.636 | 6.900 | 4.265 | 3 | 0.700 | 0.762 | 5.60 |
| 19 | 4 | 2.77 | 0.644 | 1.178 | 6.800 | 4.599 | 1 | 0.660 | 0.776 | 5.63 |
| 20 | 6 | 3.07 | 0.800 | 1.964 | 6.500 | 4.363 | 4 | 0.589 | 0.756 | 5.17 |
| 21 | 6 | 4.00 | 1.00 | 2.2 | 4.200 | 3.104 | 1 | 0.680 | 0.673 | 3.42 |
| 22 | 5 | 6.00 | 0.709 | 0.982 | 6.600 | 4.404 | 3 | 0.610 | 0.710 | 5.38 |
| Mean | 4.909 | 3.45 | 0.69 | 1.744 | 6.095 | 3.935 | 1.954 | 0.647 | 0.737 | 5.029 |
| s.d. | 1.659 | 1.085 | 0.209 | 1.25 | 0.047 | 0.034 | 0.109 | 0.005 | 0.002 | 0.542 |

## Table S7. DIYABC results for all scenarios (see Figure 3 A for graphic representation). Effective population sizes are represented by N, the first subscript represents order in time and the second represents the scenario in which the parameter was used (i.e. N_1(1,2)_ is the parameter for most recent population sizes shared in scenarios 1 and 2). Time (number of generations) was represented as t with higher values corresponding to events further in the past. Displayed values correspond to quartile 0.05 (q050), mean and quartile 0.95 (q95).

| **Parameters** | **Scenario 1** | | | **Scenario2** | | | | **Scenario3** | | |
| --- | --- | --- | --- | --- | --- | --- | --- | --- | --- | --- |
|  | *q050* | *Mean* | *q95* | | *q050* | *Mean* | *q95* | *q050* | *Mean* | *q95* |
| t_1_ | 20.4 | 177 | 429 | | 15.8 | 137 | 388 | 96.9 | 284 | 461 |
| t_2_ | 137 | 435 | 673 | | 56.1 | 302 | 631 | 240 | 475 | 677 |
| N_1(1,2)_ | 41,400 | 56,900 | 77,800 | | 45,900 | 74,500 | 97,700 | - | - | - |
| N_22_ | - | - | - | | 25,900 | 57,000 | 88,200 | - | - | - |
| N_32_ | - | - | - | | 11,300 | 28,900 | 58,700 | - | - | - |
| N_13_ | - | - | - | | - | - | - | 43,100 | 70,600 | 97,100 |
| N_23_ | - | - | - | | - | - | - | 100 | 376 | 660 |
| N_33_ | - | - | - | | - | - | - | 61,600 | 87,600 | 99400 |
| **µmic_1** | 1.03e-04 | 1.30e-04 | 1.74e-04 | | 1.36e-04 | 3.41e04 | 7.47e-04 | 6.45e-04 | 8.75e-04 | 1.00e-03 |
| **snimic_1** | 1.98e-08 | 3.79e-07 | 1.32e-06 | | 1.32e-08 | 2.09e-07 | 8.20e-07 | 1.50e-08 | 1.15e-06 | 5.89e-06 |

## Appendix S2.

**Methods for Tables S8 and S9**

The search for data to construct Tables S8 and S9 was performed using different combinations of the key words “*Quercus*”, “microsatellite*”, “genetic*” and “Oak*” in SCOPUS and ISI Web of Science. Papers were selected aiming to find case studies from oak species around the world which used a similar set of microsatellite loci markers as the one used in this study.

## Table S8. Comparison of expected heterozygosity of species of oaks studied with a similar set of nuclear microsatellites as the ones used for Q. humboldtii (see Appendix S2 for methods). Regions were grouped in four blocks which included 1: North America and North of Mexico, 2: from Mexico to Colombia, 3: Europe, 4: East of Asia

| **Block** | **Species** | **Region** | **Reference*** | **nSSR**  **loci** | **H_e_** |
| --- | --- | --- | --- | --- | --- |
| 1 | *Q. virginiana* | North America | (Cavender-Bares et al., 2011) | 11 | 0.59 |
| 1 | *Q. oleoides* | North America | (Cavender-Bares et al., 2011) | 11 | 0.71 |
| 1 | *Q. fusiformis* | North America | (Cavender-Bares et al., 2015) | 11 | 0.71 |
| 1 | *Q. geminata* | North America | (Cavender-Bares et al., 2015) | 11 | 0.73 |
| 1 | *Q. minima* | North America | (Cavender-Bares et al., 2015) | 11 | 0.76 |
| 1 | *Q. oleoides* | North America | (Cavender-Bares et al., 2015) | 11 | 0.77 |
| 1 | *Q. virginiana* | North America | (Cavender-Bares et al., 2015) | 11 | 0.8 |
| 1 | *Q. conzatii* | North Mexico | (McCauley et al., 2019) | 7 | 0.74-0.837 |
| 1 | *Q. urbani* | North Mexico | (McCauley et al., 2019) | 7 | 0.576-0.758 |
| 1 | *Q. lobata* | North America | (Gugger et al., 2013) | 7 | 0.43-0.74 |
| 1 | *Q. garryana* | North America | (Marsico et al., 2009) | 7 | 0.44-0.66 |
| 2 | *Q. oleoides (CR)* | Central America | (Cavender-Bares et al., 2011) | 11 | 0.57 |
| 2 | *Q. brandegeei* | Mexico | (Cavender-Bares et al., 2015) | 11 | 0.59 |
| 2 | *Q. oleoides (CR)* | Central America | (Cavender-Bares et al., 2015) | 11 | 0.65 |
| 2 | *Q. sagraeana* | Cuba | (Cavender-Bares et al., 2015) | 11 | 0.61 |
| 2 | *Q. conzatii* | Mexico S | (McCauley et al., 2019) | 7 | 0.588-0.784 |
| 2 | *Q. urbani* | Mexico S | (McCauley et al., 2019) | 7 | 0.541-0.706 |
| 2 | *Q. radiata* | Mexico | (McCauley et al., 2019) | 7 | 0.673-0.748 |
| 2 | *Q. tarahumara* | Mexico | (McCauley et al., 2019) | 7 | 0.69-0.745 |
| 2 | *Q. oleoides (CR)* | Central America | (Deacon & Cavender-Bares, 2015) | 11 | 0.628 |
| 2 | *Q. scytophylla x Q. sideroxyla x Q. hypoleucoides* | Mexico | (Peñaloza-Ramírez et al., 2010) | 7 | 0.55-0.84 |
| 2 | *Q. affinis x Q. laurina* | Mexico | (Ramos-Ortiz et al., 2016) | 9 | 0.75-0.83 |
| 2 | *Q. humboldtii* | North Eastern Colombia | (Fernández & Sork, 2005) | 4 | 0.84 |
| 2 | *Q. humboldtii* | North Eastern Colombia | (Fernández & Sork, 2007) | 3 | 0.813 |
| 2 | *Q, humboldtii* | Colombia | (This study) | 10 | 0.737 |
| 3 | *Q. ilex* | Europe | (Vernesi et al., 2012) | 7 | 0.626-0.639 |
| 4 | *Q. kerri* | East Asia | (Jiang et al., 2017) | 10 | 0.46-0.69 |
| 4 | *Q. phillyraeoides* | East Asia | (Harada et al., 2018) | 11 | 0.605 |
| 4 | *Q. aquifolioides* | East Asia | (Du et al., 2017) | 11 | 0.71 |
| 4 | *Q. mongolica* | East Asia | (Zeng et al., 2015) | 19 | 0.74 |
| 4 | *Q. liaotungensis* | East Asia | (Zeng et al., 2010) | 19 | 0.801 |
| 4 | *Q. crispula* | East Asia | (Ohsawa et al., 2011) | 7 | 0.688-0.756 |
| 4 | *Q. acuta* | East Asia | (Lee et al., 2010) | 15 | 0.0196-0.6458 |

***References for Table S8**

Cavender-Bares, J., Gonzalez-Rodriguez, A., Pahlich, A., Koehler, K., & Deacon, N. (2011). Phylogeography and climatic niche evolution in live oaks (*Quercus* series *Virentes*) from the tropics to the temperate zone. *Journal of Biogeography*, 38(5), 962–981. <https://doi.org/10.1111/j.1365-2699.2010.02451.x>

Cavender-Bares, J., González-Rodrı́guez, A., Eaton, D. A. R., Hipp, A. A. L., Beulke, A., & Manos, P. S. (2015). Phylogeny and biogeography of the American live oaks (*Quercus* subsection *Virentes*): a genomic and population genetics approach. *Molecular Ecology*, *24*(14), 3668–3687. <https://doi.org/10.1111/mec.13269>

Deacon, N. J., & Cavender-Bares, J. (2015). Limited Pollen Dispersal Contributes to Population Genetic Structure but Not Local Adaptation in *Quercus oleoides* Forests of Costa Rica. *PLOS ONE*, *10*(9), e0138783. <https://doi.org/10.1371/journal.pone.0138783>

Du, F. K., Hou, M., Wang, W., Mao, K., & Hampe, A. (2017). Phylogeography of *Quercus aquifolioides* provides novel insights into the Neogene history of a major global hotspot of plant diversity in south-west China. *Journal of Biogeography*, *44*(2), 294–307. <https://doi.org/10.1111/jbi.12836>

Fernández-M., J. F., & Sork, V. L. (2005). Mating Patterns of a Subdivided Population of the Andean Oak (*Quercus humboldtii* Bonpl., Fagaceae). *Journal of Heredity*, *96*(6), 635–643. [https://doi.org/https://doi.org/10.1093/jhered/esi104](https://doi.org/https:/doi.org/10.1093/jhered/esi104)

Fernández-M., J. F., & Sork, V. L. (2007). Genetic Variation in Fragmented Forest Stands of the Andean Oak *Quercus humboldtii* Bonpl. (Fagaceae). *Biotropica*, *39*(1), 72–78. <https://doi.org/10.1111/j.1744-7429.2006.00217.x>

Gugger, P. F., Ikegami, M., & Sork, V. L. (2013). Influence of late Quaternary climate change on present patterns of genetic variation in valley oak, *Quercus lobata* Née. *Molecular Ecology*, *22*(13), 3598–3612. <https://doi.org/10.1111/mec.12317>

Harada, K., Dwiyanti, F. G., Liu, H.-Z., Takeichi, Y., Nakatani, N., & Kamiya, K. (2018). Genetic variation and structure of Ubame oak, *Quercus phillyraeoides*, in Japan revealed by chloroplast DNA and nuclear microsatellite markers. *Genes & Genetic Systems*, *93*(2), 37–50. <https://doi.org/10.1266/ggs.17-00026>

Jiang, X.-L., An, M., Zheng, S.-S., Deng, M., & Su, Z.-H. (2017). Geographical isolation and environmental heterogeneity contribute to the spatial genetic patterns of *Quercus kerrii* (Fagaceae). *Heredity*, *120*(3), 219–233. <https://doi.org/10.1038/s41437-017-0012-7>

Lee, J.-H., Park, M.-H., Min, G.-S., & Choi, B.-H. (2010). Isolation and Characterization of 13 Microsatellite Loci from Korean *Quercus acuta* (Fagaceae). *Journal of Plant Biology*, *53*(3), 201–204. <https://doi.org/10.1007/s12374-010-9105-z>

Marsico, T. D., Hellmann, J. J., & Romero-Severson, J. (2009). Patterns of seed dispersal and pollen flow in *Quercus garryana* (Fagaceae) following post-glacial climatic changes. *Journal of Biogeography*, *36*(5), 929–941. <https://doi.org/10.1111/j.1365-2699.2008.02049.x>

McCauley, R. A., Cortés-Palomec, A. C., & Oyama, K. (2019). Species diversification in a lineage of Mexican red oak (*Quercus* section *Lobatae* subsection *Racemiflorae*) the interplay between distance, habitat, and hybridization. *Tree Genetics & Genomes*, *15*(2). <https://doi.org/10.1007/s11295-019-1333-x>

Ohsawa, T., Saito, Y., & Ide, Y. (2011). Multiple elevational patterns of nuclear genetic variations in oak populations elucidated by grouping populations with chloroplast markers. *Scandinavian Journal of Forest Research*, *26*(4), 305–318. <https://doi.org/10.1080/02827581.2011.570782>

Peñaloza-Ramírez, J. M., González-Rodríguez, A., Mendoza-Cuenca, L., Caron, H., Kremer, A., & Oyama, K. (2010). Interspecific gene flow in a multispecies oak hybrid zone in the Sierra Tarahumara of Mexico. *Annals of Botany*, *105*(3), 389–399. <https://doi.org/10.1093/aob/mcp301>

Ramos-Ortiz, S., Oyama, K., Rodríguez-Correa, H., & González-Rodríguez, A. (2016). Geographic structure of genetic and phenotypic variation in the hybrid zone between *Quercus affinis* and *Q. laurina* in México. *Plant Species Biology*, *31*(3), 219–232. <https://doi.org/10.1111/1442-1984.12109>

Vernesi, C., Rocchini, D., Pecchioli, E., Neteler, M., Vendramin, G. G., & Paffetti, D. (2012). A landscape genetics approach reveals ecological-based differentiation in populations of holm oak (*Quercus ilex* L.) at the northern limit of its range. *Biological Journal of the Linnean Society*, *107*(2), 458–467. <https://doi.org/10.1111/j.1095-8312.2012.01940.x>

Zeng, Y.-F., Liao, W.-J., Petit, R. J., & Zhang, D.-Y. (2010). Exploring Species Limits in Two Closely Related Chinese Oaks. *PLoS ONE*, *5*(11), e15529. <https://doi.org/10.1371/journal.pone.0015529>

Zeng, Y.-F., Wang, W.-T., Liao, W.-J., Wang, H.-F., & Zhang, D.-Y. (2015). Multiple glacial refugia for cool-temperate deciduous trees in northern East Asia: the Mongolian oak as a case study. *Molecular Ecology*, *24*(22), 5676–5691. <https://doi.org/10.1111/mec.13408>

## Table S9. Comparison of haplotype richness and genetic diversity (hS) of species of oaks studied with a similar set of chloroplast microsatellites as the ones used on Q. humboldtii (see Appendix S2 for methods). Regions were grouped in three blocks which included 1: North America and North of Mexico, 2: from Mexico to Central America, 3: Europe, 4: Asia

| **Block** | **Species** | **Region** | **Reference*** | **cpSSR**  **loci** | ***hs*** | **Haplotype**  **richness** |
| --- | --- | --- | --- | --- | --- | --- |
| 1 | *Q. conzatii* | Mexico N | (McCauley et al., 2019) | 7 | 0.051-0.06 | 16 |
| 1 | *Q. urbani* | Mexico N | (McCauley et al., 2019) | 7 | 0.051-0.286 | 13 |
| 1 | *Q. lobata* | North America | (Grivet et al., 2006) | 6 | 0.285 | 39 |
| 1 | *Q. garryana* | North America | (Marsico et al., 2009) | 5 | 0.08 | 6 |
| 2 | *Q. insiginis* | Mexico | (Rodríguez-Correa et al., 2017) | 9 | 0.42 | 28 |
| 2 | *Q. insiginis* | Central America | (Rodríguez-Correa et al., 2017) | 9 | 0.91-0.93 | 28 |
| 2 | *Q. sapotifolia* | Central America | (Rodríguez-Correa et al., 2017) | 9 | 0.79-0.88 | 34 |
| 2 | *Q. sapotifolia* | Mexico | (Rodríguez-Correa et al., 2017) | 9 | 0.64 | 34 |
| 2 | *Q. costarricensis* | Central America | (Rodríguez-Correa et al., 2018) | 9 | 0.94 | 18 |
| 2 | *Q. bumelioides* | Central America | (Rodríguez-Correa et al., 2018) | 9 | 0.72 | 26 |
| 2 | *Q. conzatii* | Mexico S | (McCauley et al., 2019) | 7 | 0.051-0.103 | 16 |
| 2 | *Q. urbani* | Mexico S | (McCauley et al., 2019) | 7 | 0.026-0.127 | 13 |
| 2 | *Q. radiata* | Mexico | (McCauley et al., 2019) | 7 | 0.051 | 10 |
| 2 | *Q. tarahumara* | Mexico | (McCauley et al., 2019) | 7 | 0.071-0.106 | 4 |
| 2 | *Q. deserticola* | Mexico | (Rodríguez-Gómez et al., 2018) | 7 | 0.754 | 54 |
| 2 | *Q. crassifolia* | Mexico | (Tovar-Sánchez et al., 2008) | 3 | 0.407-0.507 | 26 |
| 2 | *Q. crassipes* | Mexico | (Tovar-Sánchez et al., 2008) | 3 | 0.2-0.427 | 26 |
| 2 | *Q. affinis x Q. laurina* | Mexico | (Ramos-Ortiz et al., 2016) | 4 | 0.90-0.96 | 35 |
| 2 | *Q. humboldtii* | Colombia | (This study) | 9 | 0.69 | 52 |
| 3 | *Q. robur* | Europe | (Chmielewski et al., 2015) | 14 | 0.264 | 23 |
| 3 | *Q. petraea* | Europe | (Chmielewski et al., 2015) | 14 | 0.296 | 17 |
| 3 | *Q. robur* | Europe | (Neophytou & Michiels, 2013) | 10 | 0.346 | 11 |
| 3 | *Q. petraea* | Europe | (Neophytou & Michiels, 2013) | 10 | 0.162 | 12 |
| 3 | *Q. pubescens* | Europe | (Neophytou & Michiels, 2013) | 10 | 0.078 | 4 |
| 3 | European oak complex | Europe | (Grivet et al., 2006) | 6 | 0.285 | 11 |
| 3 | *Q. suber* | Europe | (Magri et al., 2007) | 14 |  | 5 |
| 4 | *Q. cerris* | Asia | (Bagnoli et al., 2015) | 6 | 0.109 | 35 |
| 4 | *Q. crispula* | Asia | (Ohsawa et al., 2011) | 6 |  | 6 |

***References for Table S9**

Bagnoli, F., Tsuda, Y., Fineschi, S., Bruschi, P., Magri, D., Zhelev, P., Paule, L., Simeone, M. C., González-Martínez, S. C., & Vendramin, G. G. (2015). Combining molecular and fossil data to infer demographic history of *Quercus cerris*: insights on European eastern glacial refugia. *Journal of Biogeography*, *43*(4), 679–690. <https://doi.org/10.1111/jbi.12673>

Chmielewski, M., Meyza, K., Chybicki, I., Dzialuk, A., Litkowiec, M., & Burczyk, J. (2015). Chloroplast microsatellites as a tool for phylogeographic studies: the case of white oaks in Poland. *iForest - Biogeosciences and Forestry*, *8*(6), 765–771. <https://doi.org/10.3832/ifor1597-008>

Grivet, D., Deguillox, M.-F., Petit, R. J., & Sork, V. L. (2006). Contrasting patterns of historical colonization in white oaks (*Quercus* spp.) in California and Europe. *Molecular Ecology*, *15*(13), 4085–4093. <https://doi.org/10.1111/j.1365-294x.2006.03083.x>

Magri, D., Fineschi, S., Bellarosa, R., Buonamici, A., Sebastiani, F., Schirone, B., Simeone, M. C., & Vendramin, G. G. (2007). The distribution of *Quercus* suberchloroplast haplotypes matches the palaeogeographical history of the western Mediterranean. *Molecular Ecology*, *16*(24), 5259–5266. <https://doi.org/10.1111/j.1365-294x.2007.03587.x>

Marsico, T. D., Hellmann, J. J., & Romero-Severson, J. (2009). Patterns of seed dispersal and pollen flow in *Quercus garryana* (Fagaceae) following post-glacial climatic changes. *Journal of Biogeography*, *36*(5), 929–941. <https://doi.org/10.1111/j.1365-2699.2008.02049.x>

McCauley, R. A., Cortés-Palomec, A. C., & Oyama, K. (2019). Species diversification in a lineage of Mexican red oak (*Quercus* section *Lobatae* subsection *Racemiflorae*) the interplay between distance, habitat, and hybridization. *Tree Genetics & Genomes*, *15*(2). <https://doi.org/10.1007/s11295-019-1333-x>

Neophytou, C., & Michiels, H.-G. (2013). Upper Rhine Valley: A migration crossroads of middle European oaks. *Forest Ecology and Management*, *304*, 89–98. <https://doi.org/10.1016/j.foreco.2013.04.020>

Ohsawa, T., Saito, Y., & Ide, Y. (2011). Multiple elevational patterns of nuclear genetic variations in oak populations elucidated by grouping populations with chloroplast markers. *Scandinavian Journal of Forest Research*, *26*(4), 305–318. <https://doi.org/10.1080/02827581.2011.570782>

Ramos-Ortiz, S., Oyama, K., Rodríguez-Correa, H., & González-Rodríguez, A. (2016). Geographic structure of genetic and phenotypic variation in the hybrid zone between *Quercus affinis* and *Q. laurina* in México. *Plant Species Biology*, *31*(3), 219–232. <https://doi.org/10.1111/1442-1984.12109>

Rodríguez-Correa, H., Oyama, K., Quesada, M., Fuchs, E. J., & González-Rodríguez, A. (2018). Contrasting Patterns of Population History and Seed-mediated Gene Flow in Two Endemic Costa Rican Oak Species. *Journal of Heredity*, *109*(5), 530–542. <https://doi.org/10.1093/jhered/esy011>

Rodríguez-Correa, H., Oyama, K., Quesada, M., Fuchs, E. J., Quezada, M., Ferrufino, L., Valencia-Ávalos, S., Cascante-Marín, A., & González-Rodríguez, A. (2017). Complex phylogeographic patterns indicate Central American origin of two widespread Mesoamerican *Quercus* (Fagaceae) species. *Tree Genetics & Genomes*, *13*(3). <https://doi.org/10.1007/s11295-017-1147-7>

Rodríguez-Gómez, F., Oyama, K., Ochoa-Orozco, M., Mendoza-Cuenca, L., Gaytán-Legaria, R., & González-Rodríguez, A. (2018). Phylogeography and climate-associated morphological variation in the endemic white oak *Quercus deserticola* (Fagaceae) along the Trans-Mexican Volcanic Belt. *Botany*, *96*(2), 121–133. <https://doi.org/10.1139/cjb-2017-0116>

Tovar-Sánchez, E., Mussali-Galante, P., Esteban-Jiménez, R., Piñero, D., Arias, D. M., Dorado, O., & Oyama, K. (2008). Chloroplast DNA polymorphism reveals geographic structure and introgression in the *Quercus crassifolia* x *Quercus crassipes* hybrid complex in Mexico. *Botany*, *86*(3), 228–239. <https://doi.org/10.1139/b07-128>

**Appendix S3**

**ENM Results**

A total of 145 records were used to construct the model. The present-day model showed a good performance according to the AUC value (0.960, s.d. 0.007). The model for the present generally agrees with the known distribution for *Quercus humboldtii.* However, it also predicts high environmental suitability for areas with no records, such as the North East of COc, the Serranía de la Macarena, the Ecuadorian Andes and the Serranía de Tabasará in Panama (Figure S1 A). Similarly, the three mid Holocene models coincide in the prediction of high environmental suitability on the three main cordilleras and low suitability in the Magdalena and Cauca valleys (Figure S1 B, C and D).

There are variations among the mid Holocene models regarding suitability of the northern Colombian Andes. MIROC and CCSM models show lower environmental suitability for the northern COc and the Serranía de San Lucas (Figure S1 B and C) in comparison with the mid Holocene MPI-ESM model (Figure S1 D). Another important difference is the lack of habitat suitability shown by the mid Holocene MIROC model on highland areas of the three cordilleras and high habitat suitability on lowland areas of the Cauca Valley (Figure S1 A).

The geographic distribution of suitable environmental conditions during the LGM, for MIROC and CCSM models was mainly concentrated southwards and in lower lands than the results obtained for the mid-Holocene or the present. Also, both models show low environmental suitability for the mountains in Panama and the northern COc (Figure S1 E and F). In contrast, the MPI-ESM model for the LGM changed very little with respect with the present day or the mid Holocene models (Figure S1 G).

**Figure S1.** *Quercus humboldtii*’s ecological niche model logistic output from MaxEnt for **A.** the present day (PD). ST = Serranía de Tabasará, SD = Serranía del Darién, SP = Serranía de Perijá, SL= Serranía de San Lucas, COc = Cordillera Occidental, COr = Cordillera Oriental, CC= Cordillera Central, and SM = Serranía de La Macarena; **B**, **C,** and **D**. mid-Holocene (MH); and **E**, **F,** and **G**. last glacial maximum (LGM). Rows show the three climatic circulation models used: **C** and **F**. MIROC; **D** and **G**. MPI-ESM; and **E** and **H**. CCSM.
